# KCTD15 deregulation is associated with alterations of the NF-κB signaling in both pathological and physiological model systems

**DOI:** 10.1038/s41598-021-97775-6

**Published:** 2021-09-14

**Authors:** Giovanni Smaldone, Luigi Coppola, Katia Pane, Monica Franzese, Giuliana Beneduce, Rosanna Parasole, Giuseppe Menna, Luigi Vitagliano, Marco Salvatore, Peppino Mirabelli

**Affiliations:** 1 IRCCS SDN, Via E. Gianturco 113, 80143 Naples, Italy; 2 Department of Pediatric Hemato-Oncology, Santobono-Pausilipon Hospital, 80129 Naples, Italy; 3 Institute of Biostructures and Bioimaging, C.N.R., Via Mezzocannone n.16, 80134 Naples, Italy

**Keywords:** Haematological cancer, Leukaemia, Acute lymphocytic leukaemia

## Abstract

Like other KCTD proteins, KCTD15 is involved in important albeit distinct biological processes as cancer, neural crest formation, and obesity. Here, we characterized the role of KCTD15 in different physiological/pathological states to gain insights into its diversified function(s). The silencing of KCTD15 in MLL-rearranged leukemia models induced attenuation of the NF-κB pathway associated with a downregulation of pIKK-β and pIKB-α. Conversely, the activation of peripheral blood T cells upon PMA/ionomycin stimulation remarkably upregulated KCTD15 and, simultaneously, pIKK-β and pIKB-α. Moreover, a significant upregulation of KCTD15 was also observed in CD34 hematopoietic stem/progenitor cells where the NF-κB pathway is physiologically activated. The association between KCTD15 upregulation and increased NF-κB signaling was confirmed by luciferase assay as well as KCTD15 and IKK-β proximity ligation and immunoprecipitation experiments. The observed upregulation of IKK-β by KCTD15 provides a novel and intriguing interpretative key for understanding the protein function in a wide class of physiological/pathological conditions ranging from neuronal development to cancer and obesity/diabetes.

## Introduction

Leukemic cells originate from the malignant transformation of undifferentiated myeloid or lymphoid hematopoietic progenitors normally residing in bone marrow^1^. Then, according to the immunological features of the malignant progenitors, acute leukemias are classified as acute lymphoblastic/lymphoid leukemia (ALL) or acute myeloid leukemia (AML). About 80% of ALL occurs in children whereas the majority of AML cases are diagnosed in adult patients^2^. The different types of leukemias are caused by either genetic or environmental alterations, although the precise molecular mechanisms underlying these heterogeneous diseases are yet to be disclosed^1,2^. In the case of ALL, it is known that genetic syndromes such as Down syndrome, Fanconi anemia, Bloom syndrome, ataxia-telangiectasia, and Nijmegen breakdown syndrome predispose to the onset of leukemia. However, in the majority of the cases, acute leukemia is a de novo disease occurring in previously healthy people. Chromosomal aberrations are the hallmark of acute leukemia but are not sufficient to generate leukemia itself. In the case of ALL, frequently found chromosomal translocations include t(12;21) (ETV6-RUNX1), t(1;19)^3^, t(9;22)(BCR-ABL1)and mixed-lineage leukemia (MLL) rearrangement^4^. Regarding AML, some of the most frequently found chromosomal translocations include t(8;21)^5^, t(15;17) (PML-RARA), t(9;22) (BCR-ABL), as well as genetic mutation for NPM-1, RUNX1 and MLL^6^. Despite the plethora of genetic alterations featuring both myeloid and lymphoid acute leukemias, it is important to consider that most of them lead to the constitutive activation of critical pathways for cell growth including Janus kinase/Signal transducers and transcription activators (JAK-STAT), Phosphoinositide-3-kinase–protein kinase B (PI3K-AKT), Ras mitogen-activated protein kinase (Ras-MAPK), Glycogen synthase kinase-3β (GSK-3β), Hypoxia-inducible transcription factor 1α (HIF-1α) and Nuclear factor-kappa beta (NF-κB) among others^4,7^.

Very recently, we have found that KCTD15, a member of the emerging family of the KCTD (potassium channel tetramerization domain) proteins^8^, is upregulated in patients and cell lines of both childhoods B-cell ALL^9^ and AML^10^. Moreover, this protein also displays a characteristic and well-defined profile of expression in peripheral blood cells (granulocytes, monocytes, and lymphocytes), thus suggesting that it plays an active role in both physiological and pathological processes, although the mechanism underlying this activity is yet to be uncovered. A survey of literature data highlights that KCTD15 and its closely related homolog KCTD1 (overall sequence identity of ~ 80%) are involved in a variety and apparently unrelated biological processes. In addition to the role that KCTD15 plays in leukemia and the maturation of blood cells, previous studies have demonstrated this protein inhibits neural crest formation by attenuating Wnt/beta-catenin pathway. Moreover, KCTD15 is implicated in medulloblastoma, obesity, and diabetes^11–13^. Literature surveys indicate that KCTD1 functions present analogies and differences with those exhibited by KCTD15^9,14–18^. Indeed, KCTD1 regulates the Wnt/beta-catenin pathway and promotes adipogenesis^15,19^. Moreover, KCTD1 missense mutations cause scalp-ear-nipple syndrome^20,21^. Collectively, these observations suggest that KCTD15/KCTD1 likely plays some basic biochemical function that has effects in different physio-pathological processes.

In the present manuscript, starting from the analysis of the transcriptome of the peripheral blood of healthy individuals and leukemia patients, we highlighted the possibility that KCTD15 could be involved in the NF-κB signaling. This hypothesis was corroborated by several experiments that demonstrate the involvement of KCTD15 into the activation of the IKβ kinase (IKK-β) enzyme complex thus initiating a cascade of events that trigger the NF-κB pathway. Not only do the present findings provide a mechanism for interpreting the role of the protein in leukemia and the physiological T-cells but they provide a novel perspective for interpreting the role of KCTD1/KCTD15 in other, apparently distant, processes.

## Results

### KCTD15 deregulation influences the NF-κB signaling

**Figure 1:**
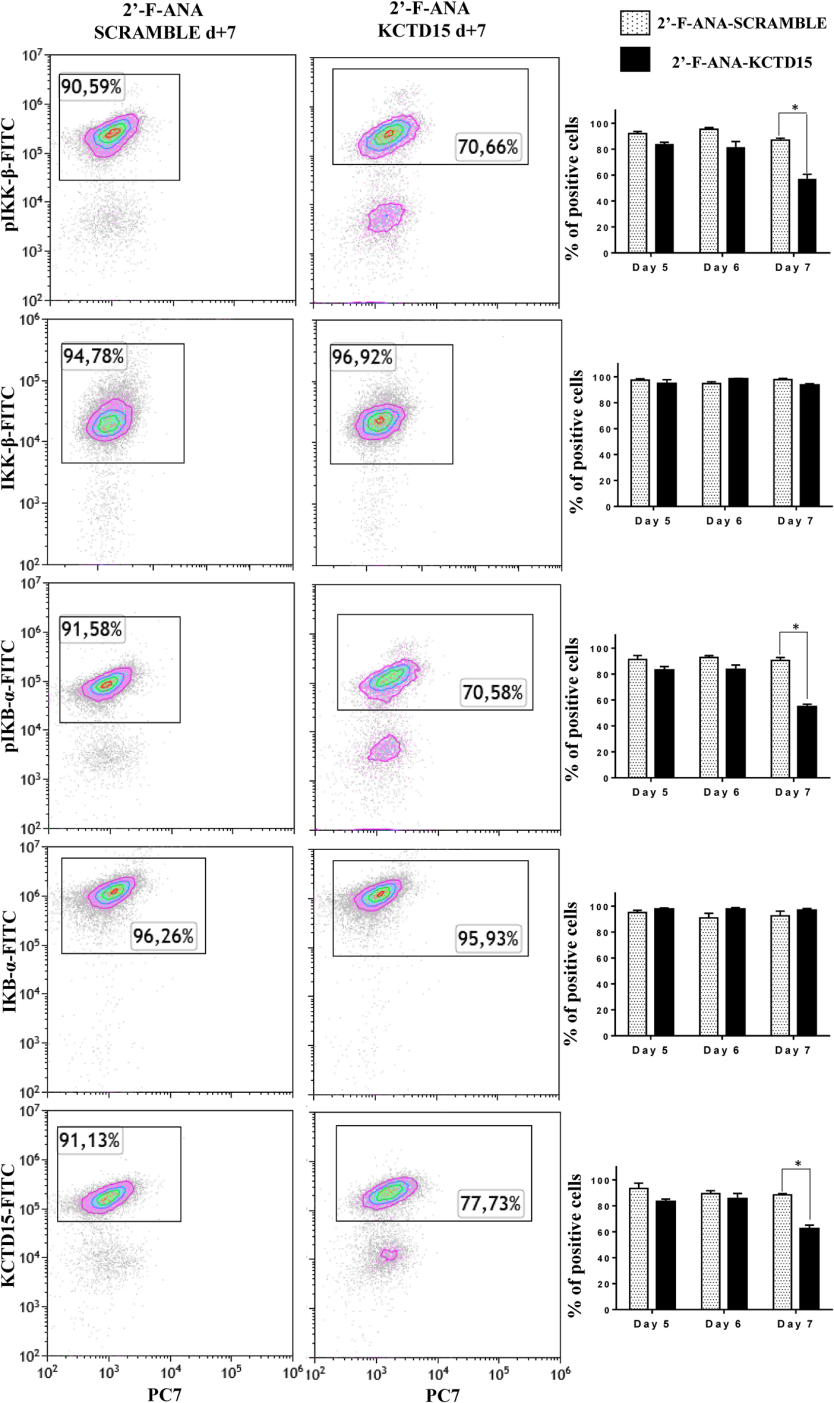
KCTD15 silencing in SEM cell lines is associated with NF-κB signaling impairment. Dot-plot analysis of NF-κB signaling proteins in 2′F-ANA KCTD15 and 2′F-ANA-Scramble treated SEM cell line at day 7 of incubation in terms of % of positive cells. Bar-histograms show the complete time-course analysis of treatment for 2′F-ANA Scramble (checkerboard bars) and 2′F-ANA KCTD15 (black bars). The % of positive cells is shown as mean ± SD of three technical independent experiments. *p-value < 0,05, Mann–Whitney *t* test.

We have recently shown that KCTD15 is strongly upregulated in B cell ALL (B-ALL), although the molecular mechanism underlying a potential action of this protein in the pathology is yet to be elucidated^9^. The involvement of KCTD15 in leukemia was initially uncovered by performing a comparative analysis of the transcriptome profile of the peripheral blood of 3 B-ALL patients and 3 healthy subjects (Bio project: PRJNA601326)^9^. To gain insights into the role played by the protein in the disease, here we re-interrogated this dataset looking at other genes that were upregulated/downregulated in this comparative analysis and clustered them according to their biological functions. In particular, the Ingenuity Pathway Analysis (IPA, release 2019) (see the “Methods” section for details) on this dataset unraveled the occurrence of a total of 873 differentially expressed genes. As shown in Supplementary Figure 1, the IPA analysis indicates that genes of the NF-κB activation pathway are the most affected ones. Indeed, 16 out of the 87 annotated genes within IPA Knowledgebase for this pathway were shown to be differently expressed by applying stringent statistical criteria. This finding is not surprising since the NF-κB pathway is frequently found to be hyper-activated in ALL^22–26^. This very preliminary observation prompted us to set up experiments aimed at evaluating the possible role of KCTD15 in the NF-κB pathway. To this scope, we decided to silence the *kctd15* gene in SEM cells, a B-cell precursor cell line established from the peripheral blood of a 5-year-old girl at B-ALL relapse and featured by the t(4;11) KMT2A-AFF1 (MLL-AFF1; MLL-AF4) translocation^27^. In this pathological model system, we tested the expression levels of the proteins associated with the NF-κB signaling after KCTD15 silencing using the 2′F-ANA methodology (see “Methods” for details). In particular, we performed flow cytometry (FCM) experiments to evaluate the expression levels of phosphorylated IKB-α (pIKB-α) and IKK-β (pIKK-β) (Fig. 1). Notably, we found a significant reduction of the proteins that play key roles in NF-κB signaling, such as of pIKK and pIKB-α at day 7 of the silencing. It is important to note that these analyses were performed specifically on live cells selected according to the FSC and SSC parameters. This selection was essential considering the significant cell mortality observed at day 7 (Ref.^9^ and Supplementary Figure 2). On the other hand, the unphosphorylated forms of these proteins were unchanged (Fig. 1), suggesting that their reduced phosphorylation does not have the time to impact their total content. The association between KCTD15 downregulation and NF-κB signaling reduction was also explored in the RS4;11 cell line, a second B-cell precursor featured with KMT2A-AFF1 (MLL-AFF1; MLL-AF4) fusion gene^27^. Also in this model system, the downregulation of KCTD15 is associated with reduced expression levels of the proteins involved in NF-κB signaling (Supplementary Figure 3). Again, considering the significant cell death in lymphoid cell lines induced by KCTD15 signaling (Ref.^9^ and Supplementary Figure 2), the overexpression levels of these proteins were specifically measured on live cells.

**Figure 2:**
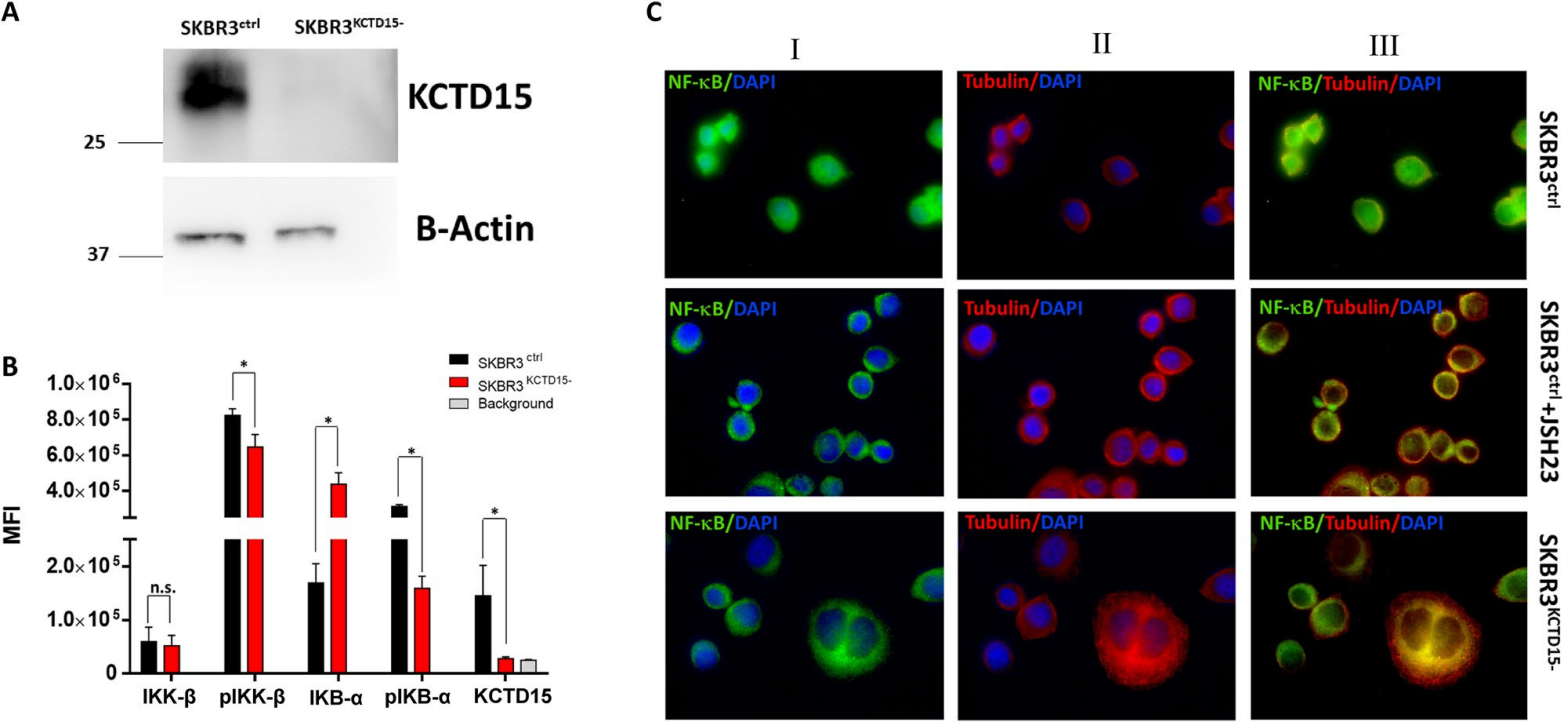
KCTD15 silencing in SKBR3 causes the inactivation of the NF-κB pathway. (**A)** Western blot analysis of KCTD15 expression in SKBR3^ctrl^ and SKBR3^KCTD15−^ obtained by CRISP/CAS9 technology. B-Actin was used as the internal control. Numbers represent molecular weight protein markers. (**B**) Bar-plot diagram of NF-κB proteins pathway in SKBR3^ctrl^ (black bars) and SKBR3^KCTD15−^ (red bars) reported as Mean Fluorescence Intensity of two independent experiments determined by cytofluorimeter. (**C**) Immunofluorescence experiments of untreated SKBR3^ctrl^ (upper panels), JSH-23 treated (middle panels) and SKBR3^KCTD15−^ (lower panels). Fluorescence microscopy experiments. Column (I) Nuclei staining with DAPI (blue) and FITC- NF-κB RelA (green). Column (II) Nuclei staining with DAPI (blue) and PE-B-tubulin (red). (III) Overlapping of FITC, PE, and DAPI channels. Magnification × 63. Scale bars 20 µm.

To overcome some limitations of these silencing analyses, as the relatively long time taken to obtain measurable effects (7 days for SEM and 13 days for RS4;11) and the significant cell lethality, and to corroborate/extend these observations, we performed KCTD15 silencing experiments in another model system using a different methodology. In particular, we selected SKBR3 cells, an ER-negative and HER-2+ breast cancer model system, featured by increased NF-κB signaling activity^28^ in which we recently found an upregulation of KCTD15 (Smaldone et al. under review) (see also Supplementary Figure 4). In SKBR3 cells, the successful inactivation of KCTD15 gene by CRISP/CAS9 recombinase (Fig. 1A) did not influence the cellular viability (Supplementary Figure 5). Interestingly, as for leukemia cell lines, this KCTD15 inactivation caused a decrease of the expression levels of both pIKB-α and pIKK-β compared to the controls. In this case, we also observed a global increase of IKB-α as a consequence of its reduced phosphorylation and, likely, degradation (Fig. 2B).

As these findings suggest that the KCTD15 downregulation could lead to a decrease of the NF-κB signaling, its status was monitored by evaluating the localization of Rel-A, a subunit of NF-κB, in SKBR3 cells. In these cells, in which the pathway is activated, Rel-A was localized in the cytoplasm and, partly, in the nucleus (Fig. 2C). However, upon KCTD15 silencing (SKBR3^KCTD15−^ cells), we observed a primary cytoplasmic localization of Rel-A in a manner that is similar to that observed after the treatment of these cells with JSH-23 (Fig. 2C), a potent inhibitor of the NF-kB nuclear translocation^29^. Of note, the inhibition of NF-κB shuttling, associated with KCTD15 downregulation, was also detected in SEM and RS4;11 cells (Supplementary Figure 6). Indeed, in these cell lines, it was possible to observe an accumulation of NF-κB in the subtle cytoplasmic rim of the two-cell line after their treatment with 2′F-ANA-KCTD15. These findings clearly indicate that KCTD15 downregulation impairs the NF-κB signaling, likely though the downregulation of pIKK-β, the active form of the kinase.

**Figure 3:**
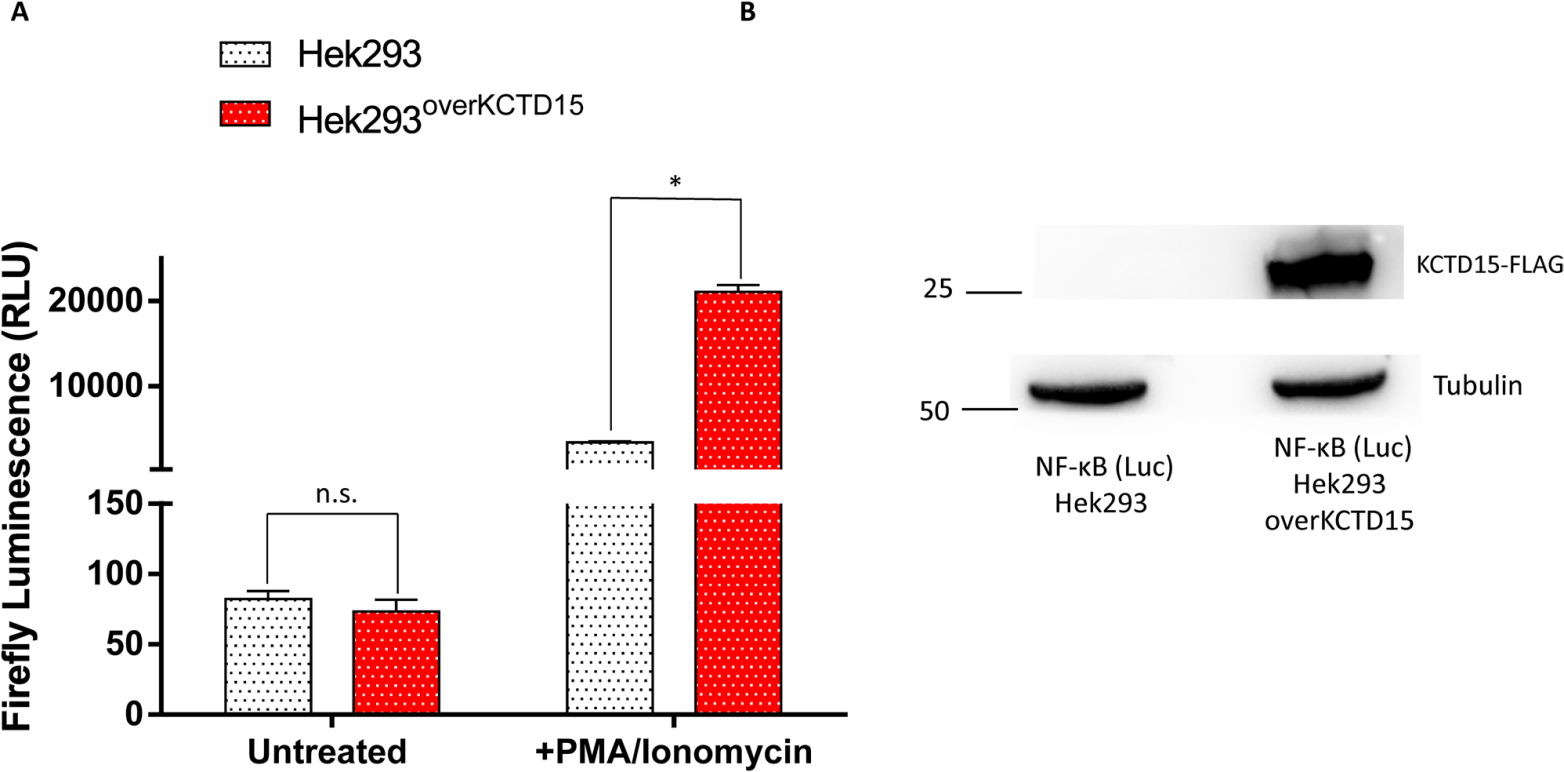
KCTD15 over-expression is associated with the hyper-activation of the NF-κB pathway. (**A**) Bar-plot diagram of firefly luciferase signal in NF-κB reporter (Luc)-HEK293 control (white dotted bars) and in NF-κB reporter (Luc)-HEK293 over-expressing KCTD15 (red dotted bars). Errors represent ± SD of three independent experiments. *p-value < 0.05, Mann–Whitney *t* test. (**B**) Western blot analysis of NF-κB reporter (Luc)-HEK293 control and NF-κB reporter (Luc)-HEK293 over-expressing KCTD15-FLAG. B-tubulin was used as an internal control. Numbers represent molecular weight protein markers.

The association between KCTD15 expression levels and NF-κB signaling was also monitored in an additional model system, the NF-κB reporter (Luc)-HEK293 cell line, that produces the luciferase enzyme under the control of NF-κB transcription factor after its stimulation with either TNF-alpha or PMA/Ionomycin. As shown in Fig. 3, we found that the transient over-expression of KCTD15 did not affect the luciferase activity. However, when cells are stimulated with PMA/Ionomycin the transient over-expression of KCTD15 increased the luciferase activity.

### KCTD15 is important for the activation of NF-κB in physiological contexts

**Figure 4:**
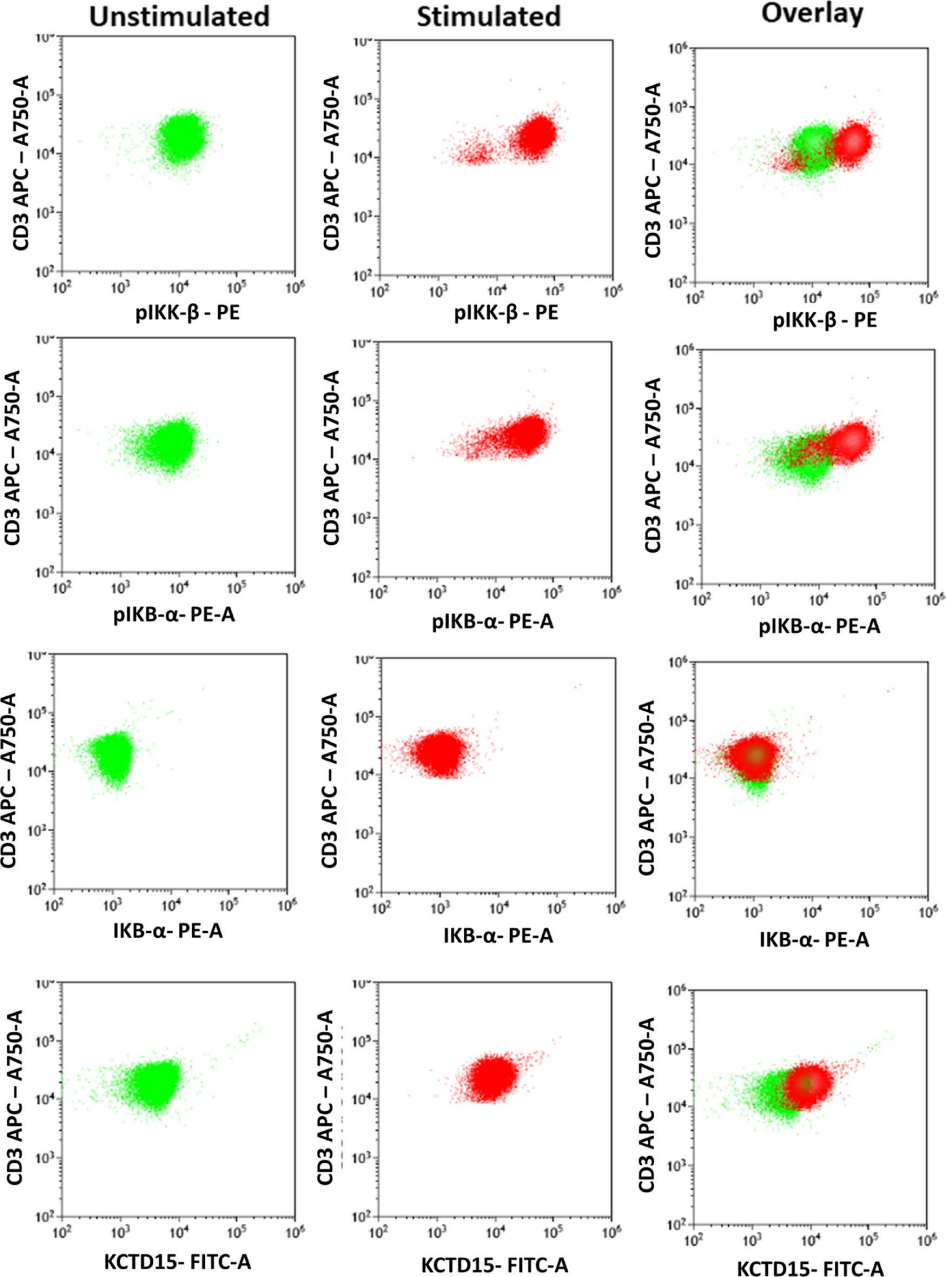
Stimulation of circulating lymphocytes with PMA/Ionomycin is associated to KCTD15 upregulation. Overlay dot-plot showing the higher levels of pIKK, pIKB-α, and KCTD15 in PMA/Ionomycin activated lymphocytes (red) compared to unstimulated lymphocytes (green). IKB-α protein remains unchanged. Results are representative of two independent experiments.

The functional role of KCTD15 in conjunction with the NF-κB pathway was additionally investigated in physiological contexts using peripheral blood circulating lymphocytes and CD34 positive hematopoietic progenitors. Specifically, in the case of PB lymphocytes, the NF-κB pathway is critical for the activation of T cells in response to external stimuli for the production of proinflammatory cytokines, such as TNF-α. To evaluate the role, if any, that KCTD15 could play in the early stages of T-cells activation, we stimulated peripheral blood T-cells for 3 h using Duractive™ activation tubes containing an activation mix composed of phorbol 12-myristate 13-acetate (PMA), Ionomycin, and Brefeldin A. The mix is specifically designed for inhibiting cytokine degranulation. As expected, the application of the protocol induced the production of TNF-α cytokine in stimulated T-lymphocytes (Supplementary Figure 7). Since the production of the cytokine in these lymphocytes is induced by the canonical NF-κB signaling, we monitored the phosphorylation levels of IKK-β, and IKB-α and non-phosphorylated form of IKB-α before and after the stimulation. As shown in Fig. 4, the addition of the mix upregulated both pIKK-β and pIKB-α. This observation corroborates the notion that the stimulation occurs through the activation of the NF-κB signaling. Interestingly, the analysis of the KCTD15 levels in response to the PMA/Ionomycin activation highlights a clear upregulation of the protein (Fig. 4).

**Figure 5:**
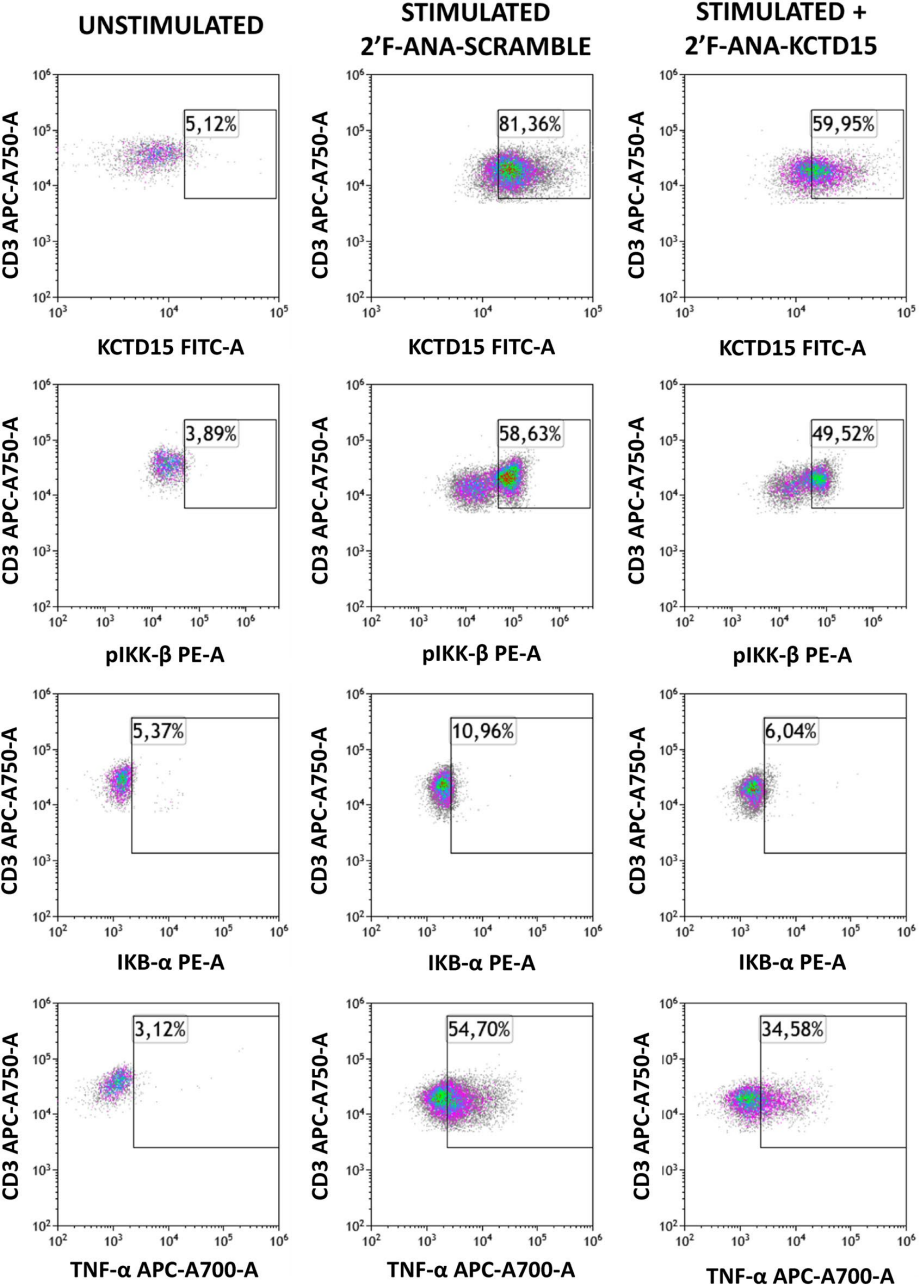
Inhibition of KCTD15 translation impairs TNF-alpha production in PMA/Ionomycin stimulated lymphocytes: density-plot analysis of Unstimulated (left), PMA/Ionomycin stimulated plus 2′F-ANA-Scramble (middle) and 2′F-ANA-KCTD15 (right) PBMCs. Numbers represent the % of the positive cells. Results are representative of two independent experiments.

**Figure 6:**
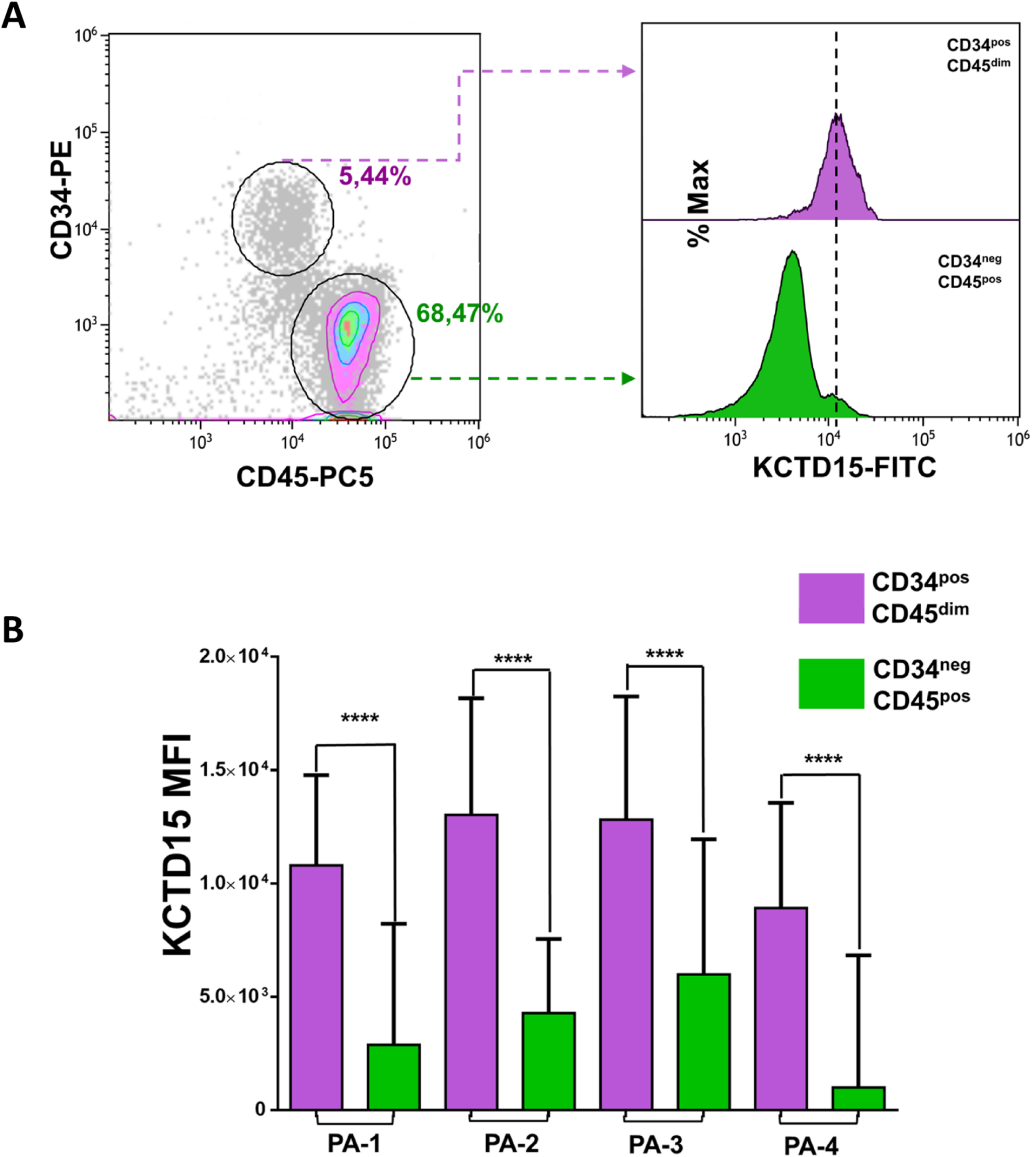
KCTD15 upregulation in hematopoietic stem cells. (**A**) Contour with density plot showing the selection of hematopoietic stem cells (CD34^pos^/CD45^dim^ events) and mature cells (CD34^neg^/CD45^pos^ events) in density gradient purified BM cells from a B-ALL patient after therapy at day + 78. Numbers are referred to as the percentage of gated cells. (**B**) Bar-plot displaying KCTD15 fluorescence intensity (in term of mean of fluorescence and SD) in CD34^pos^/CD45^dim^ (violet) and CD34^neg^/CD45^pos^ (green) (****p < 0.0001, unpaired *t* test). The error bar represents SD.

Successively, to evaluate the possible role of KCTD15 in the signal transduction for the production of the TNF-alpha cytokine, we decided to transiently inhibit KCTD15 mRNA with 2′F-ANA antisense nucleotides. To this aim, Peripheral Blood Mononuclear Cells (PBMCs) were pulse chased overnight with anti 2′F-ANA-KCTD15 oligo at + 4 °C for allowing cellular uptake. The day after, unstimulated lymphocytes, were used to define the gate boundary and to determine the percentages of antigen positivity. The treatment of PBMC with the 2′F-ANA-KCTD15 oligo was able to moderately reduce the upregulation of KCTD15 after 3 h of PMA-Ionomycin stimulation (Fig. 5). While no significant differences were detected for IKB-α expression, the KCTD15 reduction was associated with lower TNF-alpha (34.58% vs 54.70%) and pIKK-β (49.52% vs 58.63%) production compared to the controls. Finally, since NF-κB activation is physiologically important in the ontogenesis of hematopoietic stem cells (HSC)^30^, we decided to investigate KCTD15 expression levels in this cellular compartment. To this aim, we selected the CD34 positive cells from cryopreserved BM samples of four pediatric B-ALL patients with undetectable minimal residual disease (day + 78 of chemotherapy treatment) and analyzed them by FCM. Remarkably, we found that KCTD15 levels were significantly higher in CD34^pos^/CD45^dim^ hematopoietic stem cells when compared to mature lymphocytes (Fig. 6, and Supplementary Figure 8). Once again, these findings highlight a close connection between KCTD5 upregulation and NF-κB activation and suggest that KCTD15 could be important for sustaining the NF-κB signaling during the development of hematopoietic progenitors in physiological conditions.

### KCTD15 as a novel interactor of IKK-β

**Figure 7:**
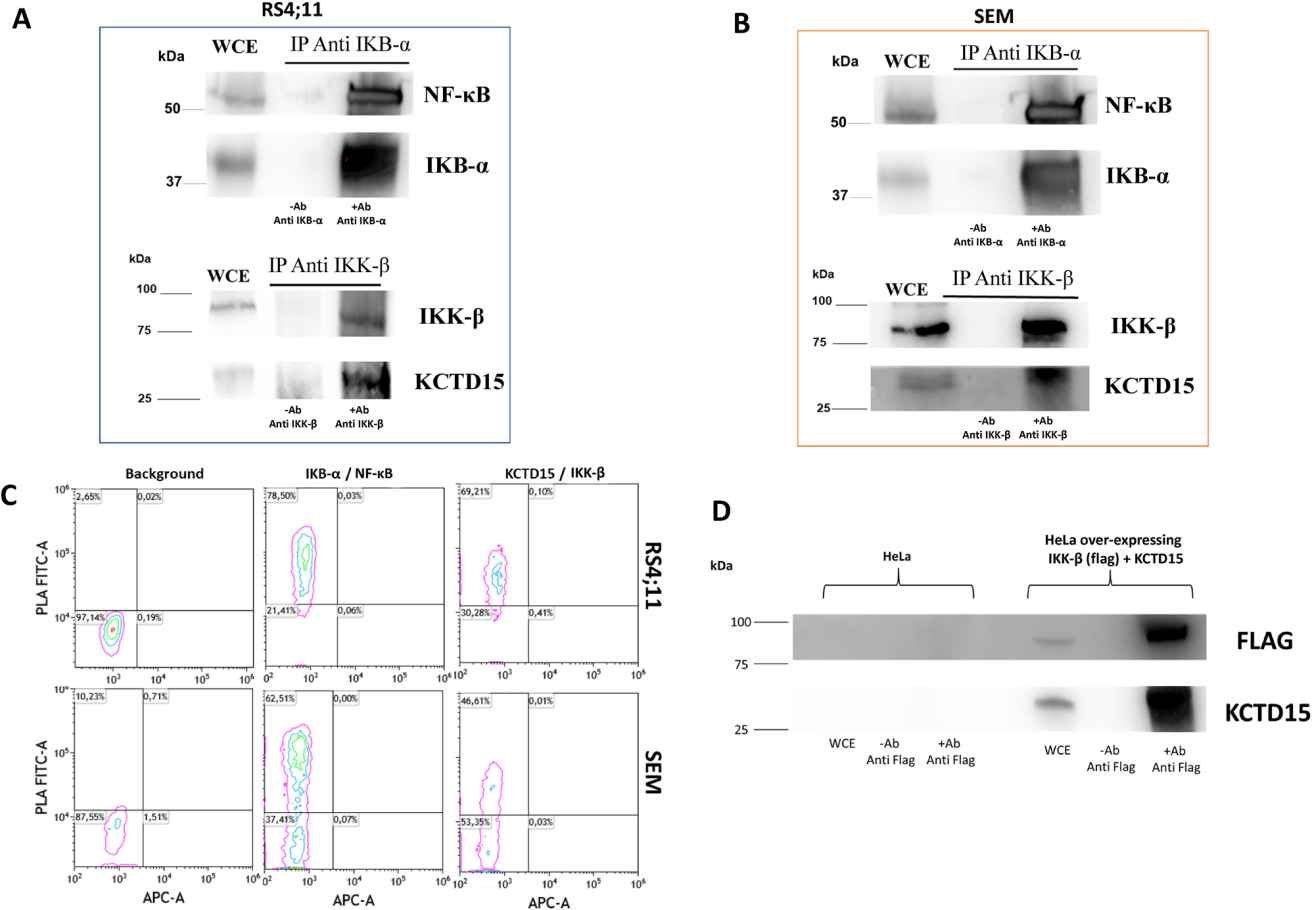
KCTD15 could be a novel interactor of IKK-β. RS4;11 (**A**) and SEM (**B**) cell extracts were incubated with antibody against IKK-β and IKB-α, precipitated by Protein A/G beads and detected by Western blots using a reported antibody. (+ Ab Anti #) = elution step in the presence of antibodies. (− Ab Anti #) = elution without antibody. *WCE *whole cell extract. The number represents the molecular weight of the protein marker expressed in kDa. (**C**) PLA experiments were conducted on active growing RS4;11 and SEM cells. PLA signal associated with FITC fluorescence was recorded using a Cytoflex cytometer. Negative technical control (Background column) was obtained using the Anti KCTD15 antibody and the two PLA probes. Numbers show the percentage of positive cells with respect to the negative control. Experiments were repeated twice with similar results. (**D**) HeLa over-expressing FLAG-IKK-β and KCTD15 cell extracts were incubated with anti-FLAG antibody, precipitated by Protein A/G beads, and detected by Western blots using the reported antibody. (+ Ab Anti #) = elution step in the presence of antibodies. (− Ab Anti #) = elution without antibody. *WCE* whole cell extract. The numbers represent the molecular weight of the protein marker expressed in kDa.

Considering the association between KCTD15 deregulation and the NF-κB signaling highlighted in the above sections, we wondered whether the KCTD15 expression could be under the control of this transcription factor. In particular, we performed an in-silico analysis for predicting NF-κB binding sites (TFBSs) in KCTD15 promoter gene, at 500 bp and 1000 bp upstream transcription start position (TSS), by Contra V3 software. According to Supplementary Figure 9, we found two possible NF-κB binding sites, such as: NFKB1 JASPAR_CORE_2016, MA0105.4 (IC 14.2, consensus: AGGGGAWTCCCCT) and MA0778.1 (IC11.4, consensus: AGGGGAWTCCCCY), with an information content (IC) of the positional weight matrices (PSWs) higher than 5 (ranging from 5 to 35). However, no any predicted NF-κB binding sites on KCTD15 promoter were statistically significant (q > 0.25). This finding makes unlikely a NF-κB RelA regulation on KCTD15 at transcriptional level. In this scenario, we evaluated the possibility of physical interaction between KCTD15 and the NF-κB activator IKK-β. To this aim, we performed the experiment presented in Fig. 7 where IKK-beta-protein immunoprecipitation in RS4;11 and SEM were screened for KCTD15 association. According to the WB analysis, we found that the immunoprecipitation for IKK-β was able to enrich the signal for the endogenous KCTD15 protein. This enrichment is qualitatively similar to that observed for NF-κB after protein immunoprecipitation for IKB-α (Fig. 7A,B—upper lane). Moreover, according to Fig. 7C, a significant PLA fluorescence signal corresponding to the endogenous partners IKK-β/KCTD15 was detected by flow cytometry. Although slightly smaller, the percentage of positive cells detected for the IKK-β/KCTD15 (69.2 and 46.6% in RS4;11 and SEM, respectively) was not very different from that observed for NF-κB/IKB-α (78.50 and 62.51% in RS4;11 and SEM, respectively), which are well known functional partners 28 and then used as a positive control in these experiments. To further validate the data obtained using the endogenous proteins, we decided to test the IKK-β /KCTD15 interaction in the HeLa cells transiently transfected with IKK-β (FLAG conjugated) and KCTD15 (at 24 h). As shown in Fig. 7D, immunoprecipitation of IKK-β-FLAG protein was significantly enriched for the KCTD15 when compared to non-transfected HeLa cells. Collectively, these results provide a strong indication that KCTD15 interacts with IKK-β or with the IKK complex.

## Discussion

The NF-κB signaling is fundamental in a plethora of physiological and pathological manifestations. In its canonical version, the activated (phosphorylated) kinase IKK phosphorylates the NF-κB inhibitor marking it for degradation^31^. These events liberate the NF-κB transcription factor that can translocate to the nucleus where it induces the transcription of a myriad of genes including anti-apoptotic proteins (cFLIP, BCL-2, and BCL-xL), growth factors, cytokines (IL-1 and IL-6), cell adhesion molecules, and chemokines^32^. In this scenario, it is not surprising that the NF-κB signaling is crucial in a countless number of biological processes that span from inflammation and immune response to cell growth/survival and development^33,34^. A full understanding of the many factors that can regulate this pathway may have immense implications for the development of innovative therapeutic applications^35^.

Starting from our recent observation that KCTD15 is upregulated in both B-ALL^9^ and AML cell lines^10^ and patient-derived samples, we here found an intriguing link between the upregulation of the protein and the activation of the NF-κB pathway that was observed in different contexts and conditions. In a first instance, the transient silencing of KCTD15 in MLL-rearranged leukemia model systems (RS4;11 and SEM) induced cell death and apoptosis with a significant downregulation of pIKK-β, the kinase deputed to the activation of the NF-κB pathway^9^. Here we initially demonstrated a down regulation of proteins involved in the NF-kB pathway upon silencing in these leukemia models systems. This finding is particularly important in light of the observations of Kuo et al. proving that in MLL-rearranged leukemias the cellular survival and proliferation were constitutively dependent by the NF-κB pathway activation^27^. An extension of these results was achieved by silencing KCTD15 in breast cancer cell line (SKBR3), where this protein is remarkably upregulated (Supplementary Figure 4). In this case, we applied the CRISP/CAS9 methodology and were able to obtain viable cells in which KCTD15 was knocked down. Despite the remarkable differences between SKBR3 and RS4;11/SEM model systems as well as the methodologies applied, we essentially replicate the results obtained in leukemia cells by measuring a reduction of the phosphorylated forms of IKK-β and IKBA. Notably, in both leukemia and breast cancer model systems, the downregulation of this key players in the NF-κB signaling was also associated with an increased cytoplasmic localization of the transcription factor where it is generally inactivated by IKBA. The association of KCTD15 deregulation and NF-kB signaling was here detected in a variety of other contexts as for example the NF-κB reporter (Luc)-HEK293 cell line^36^ and circulating T-lymphocytes upon stimulation with PMA/Ionomycin. The PMA/Ionomycin activation in T-cell bypasses the T cell membrane receptor complex and will leads to activation of several intracellular signaling pathways, which favor the production of a variety of cytokines, including the TNF-alpha that is under the transcriptional control of NF-κB^37^. As expected, the stimulation with the PMA/Ionomycin mitogen led to an increased phosphorylation of IKBA and IKK as well as production of the TNF-alpha cytokine that was concomitant with a remarkable KCTD15 upregulation. Moreover, the silencing of KCTD15 in these cells led to a reduction of TNF-alpha production. The possible link between KCTD15 expression and NF-κB signaling in normal immune/hematopoietic cells was also explored taking into consideration the CD34 compartment. Indeed, in CD34 HSPC the activation of NF-κB signaling has a positive regulation of the transcription of genes involved in the maintenance and homeostasis^38–40^. Once more, we found a significant upregulation of KCTD15 in the CD34 positive compartment that is suggestive of a possible role of KCTD15 in the physiology of HSPC mediated by the NF-κB activation.

This converging indications of a functional link between the upregulation of KCTD15 and the upregulation of the NF-κB pathway both in physiological and pathological states are corroborated at the molecular level by the observation of the physical interaction of KCTD15 and IKK-β as highlighted by PLA and immune-precipitation analyses. It is worth mentioning, however, that, although the picture that emerged from these results fits in the canonical view of an NF-κB activation by KCTD15 that favors the dissociation of the cytoplasmatic NF-κB/IKB-α complex through the upregulation of pIKK-β, it cannot be excluded that KCTD15 influences IKK-β also in other compartments. Specifically, according to the human protein atlas (https://www.proteinatlas.org/search/kctd15) KCTD15 can be found at the nuclear level too. This consideration could be of relevance according to recent findings by Armache et al. showing the ability of IKK-alpha to phosphorylate Histone H3.3 and enhance stimulation-induced transcription^41^. It cannot be excluded that in pathological and physiological model systems different from those considered in this paper, KCTD15 could interact with proteins of the IKK complex also in the cell nucleus^42^.

The association of KCTD15 with the NF-κB pathway opens a new perspective for the interpretation of the mechanism of action of KCTD15 in diversified biological contests. It has been recently reported that IKK-β is a β-catenin kinase as it phosphorylates the degron motif of β-catenin to prime it for ubiquitination/degradation mediated by the E3 ubiquitin ligase β-transducin repeat-containing protein β-TrCP. Therefore, based on the present results, the upregulation of KCTD15 could downregulate the β-catenin thus decreasing the Wnt/β-catenin pathway. This hypothesis perfectly fits with the observation that KCTD15 inhibits neural crest formation by attenuating Wnt/β-catenin signaling^43,44^. The upregulation of IKK-β is a property that KCTD15 likely shares with the close homolog KCDT1 that also suppresses the canonical Wnt/β-catenin pathway by enhancing β-catenin degradation through β-TrCP^19^. By extending these considerations, it also possible to make a tentative but intriguing functional link between KCTD15 and obesity whose connection has been found at the genetic level^43–45^. Indeed, it has been reported that IKK-β as critical for adipocyte survival and adaptive adipose remodeling in obesity^45^ and that it could serve as a key molecular switch that triggers the adipogenic differentiation of mesenchymal stem cells^46^.

The ability of KCTD15, emerged from the present analyses, play a role in the NF-κB pathway in both pathological and physiological contexts holds interesting implications on the etiology of leukemia that could also apply to other carcinogenic processes. Present findings also highlight hitherto unknown functionalities of this protein that may be shared by the other members of the KCTD family. Finally, the observed upregulation of IKK-β by KCTD15 provides a novel and intriguing interpretative key for understanding a wide and diversified class of physiological and pathological states ranging from neuronal development to obesity and diabetes. Studies aimed at putting these considerations on more solid grounds are in progress.

## Methods

### Study population

The procedures followed in the present study are in line with the Helsinki declaration and have been approved by the local ethical committees of the IRCCS-SDN (Comitato Etico IRCCS Pascale, Naples Italy—protocol number 6/16 of the 14/09/20169 and the AORN Santobono-Pausilipon (Comitato Etico Cardarelli/Pausilion, Naples Italy—protocol number 94 of 08/02/2017). Informed consent was signed by both parents (for children) or by the patient itself (for adults). The CD34 analysis was conducted at day + 78 after the completion of the therapeutic protocol on BM cells from patients coded as PA-1, PA-2, PA-3, and PA-4. These four patients were treated according to AIEOP BFM LAL 2009 protocol (ClinicalTrials.gov Identifier: NCT01117441) or subsequent guidelines (AIEOP BFM LAL 2017).

### Patient samples

Bone Marrow MonoNuclear Cells (BM-MNC) derived from pediatric B-cell Acute Lymphoblastic Leukemia (B-ALL) patients or healthy subjects were obtained by density gradient centrifugation (Pancoll^®^ density 1077 g/L, PanBiotech, Aidenbach, Germany) at 400×*g* and stored by the Biobank of SDN institute^47^ vapor phase of liquid nitrogen until the use. PBMCs were obtained from fresh venous blood collected in 3 mL EDTA vacutainer tubes (Becton Dickinson, CA, USA, Catalog. #367835) from a volunteer. In all cases about 1 × 10^6^ total live cells were obtained with a purity greater than 90% as assessed by FCM.

### Cell lines

RS4;11,SEM, SKBR3, HeLa and NF-κB reporter (Luc)-HEK293 cell lines were used for the present study. NF-κB reporter (Luc)-HEK293 cell line was purchased from BPS Bioscience (#60650) while the other model systems were authenticated at DSMZ for short tandem repeat (STR) profile. For RS4;11 and SEM culture media (Sigma-Aldrich, MO, USA) was composed of Iscove’s Modified Dulbecco’s Medium supplemented with 2 mmol/L l-Glutamine (Sigma-Aldrich) and 10% heat-inactivated FBS (ThermoFisher, GIBCO). For HeLa and NF-κB reporter (Luc)-HEK293 culture media were Dulbecco’s Modified Medium (DMEM, Gibco) supplemented with 2 mmol/L l-Glutamine (Sigma-Aldrich) and 10% heat-inactivated FBS (ThermoFisher GIBCO). For SKBR3 culture medium was McCoy’s (Gibco) supplemented with 2 mmol/L l-Glutamine (Sigma-Aldrich) and 10% heat-inactivated FBS (ThermoFisher, GIBCO). All cell lines were cultured at 37 °C in a humidified atmosphere with 5% CO_2_. Mycoplasma contamination was routinely (monthly) checked using the PCR Mycoplasma Detection KITfrom ABM (Richmond, BC, Canada).

### Generation of SKBR3^KCTD15−^ cell line

The KCTD15 silenced SKBR3 cell line was obtained using the CRISP/CAS9 technology. In brief, SKBR3 cells were transfected with KCTD15 double nickase plasmid (sc-407663-NIC-2, Santa Cruz Biotechnology, Inc. USA) to obtain KCTD15 silenced clones (SKBR3^KCTD15−^). In addition, to generate control cells (SKBR3^ctrl^), SKBR3 cells were transfected with CRISP/CAS9 control plasmid (sc-418922, Santa Cruz Biotechnology, Inc. USA) plus pReceiver-M94 plasmid (GeneCopeia, USA). Transfections were conducted using Lipofectamine 3000 reagent (L3000001, Thermo Fisher Scientific, USA) following manufacturer instructions.

### KCTD15 silencing by 2′-Deoxy, 2′Fluroarabino Nucleic Acids (2′F-ANAs) oligonucleotides

For KCTD15 silencing we used the 2′-deoxy-2′-fluoro-beta-d-arabinonucleic acid (2′F-ANA) modified oligonucleotides (ASOs)^48,49^. RS4;11 and SEM cell lines were seeded in complete media at 5 × 10^5^ cells/mL supplemented with 8 µM of a 2′F-ANA targeting the KCTD15 mRNA (2F′-ANA-KCTD15). PBMCs were isolated from healthy donors and incubated (5 × 10^5^ cells/mL) in serum free medium (Optimem, GIBCO) with 8 µM of a 2′F-ANA targeting the KCTD15 mRNA (2F′-ANA-KCTD15) for 16 h at 4 °C. Experiments using a scrambled sequence of 2′F-ANA-KCTD15 were systematically used as negative controls (2F′-ANA-scrambled). Cells were harvested at different time points of incubation (up to 13 days for RS4;11 and up to 7 days for SEM) to check KCTD15, pIKK-β S176/177, IKB-α, and pIKB-α S32/36 (H.709.9 Thermo Scientific, USA) expression. Cell viability was also evaluated by flow cytometry (FCM) using the ANNEXIN V—FITC Kit—Apoptosis Detection Kit (IM3546, Beckman Coulter, USA).

### Stimulation assays

For PBMCs experiments cells treated with 2′F-ANA targeting the KCTD15 and the scramble counterpart were harvested and added to DURActive 1 tube (C11101, Beckman Coulter). Stimulation was conducted following manufacturer instructions. For NF-κB reporter (Luc)-HEK293, 2 × 10^5^ cells/mL (6 well plate) and 2 × 10^4^ (96-well plate) were seeded in complete medium for 16 h at 37 °C. The day after cells were transfected with KCTD15-FLAG plasmid for 48 h using Lipofectamine 3000 (L3000001, Thermo Fisher Scientific) following manufacturer instructions. After 48 h of over-expression cells were treated with complete medium supplemented with 200 ng/mL Phorbol myristate acetate (PMA) and 50 ng/mL Ionomycin for 3 h at 37 °C. Subsequently, cells were treated with ONE-Step™ Luciferase Assay System (BPS-60690-2, Vinci Biochem) to detect luciferase activity using VictorNivo (Perkin Elmer, UK).

For lymphocytes stimulation assay 5 × 10^5^ PBMCs were added to DURActive 1 tube (C11101, Beckman Coulter). Stimulation was conducted following manufacturer instructions. After 3 h of incubation at 37 °C, cells were fixed and permeabilized using the PerFix Expose Kit. Incubation with antibodies was performed for 30 min at dilution 1:50 in Buffer 3 reagent using the unconjugated mouse-anti-KCTD15 antibody, unconjugated rabbit-anti-pIKK-βS176/177 antibody (J.10.3, Thermo Scientific, USA), unconjugated mouse-anti-pIKB-α S32/36 antibody (6H4L6, Thermo Scientific, USA), and anti-PE-IKB-α antibody (3D6C02, Sony, USA). After 2 wash steps, the cells were incubated again in Buffer 3 with anti-mouse secondary FITC conjugated antibody (for KCTD15 and pIKB-α S32/36) and with anti-rabbit PE (for pIKK-β) conjugated antibody. Subsequently, to evaluate surface and intracellular antigens expression, a mix of directly conjugated antibodies was added [anti CD3 (CD3-APC750, A91680 Beckman Coulter), TNF-α (TNF-α-APC700, B76295 Beckman Coulter), and CD45 (CD45-KO, B36294 Beckman Coulter)].

### Western blot assays

Lysates from human RS4;11, SEM, HeLa, SKBR3, and NF-κB reporter (Luc)-HEK293 cell lines (50 µg of protein extracts) were analyzed by Western Blot to check the protein expression. Antibodies used were: anti KCTD15 (GTX50002, Genetex International, USA), anti pIKK-βS176/177 J.10.3, Thermo Scientific, USA), anti IKB-α (662402, Biolegend, USA), Anti NF-κβ (622602, Biolegend, USA), anti β-actin (ab11004, Abcam, UK) and anti β-tubulin (Sigma-Aldrich Cat# T0198) as internal controls. Proteins were acquired using the ChemiDoc Imaging System (Bio-Rad, USA) coupled with Image Lab software. Protein normalization was conducted using the Stain-Free technology^50,51^ (Biorad, USA).

### Immunoprecipitation experiments

For endogenous immunoprecipitation analyses 2 mg of RS4;11, SEM cell lysates were incubated with 2 μg/mL of anti-IKK-β (GTX107970, Genetex International, USA) and IKβ-α (662402, Biolegend, USA) antibodies for 4 h at 4 °C. For recombinant immunoprecipitation analysis, 1.8 × 10^6^ HeLa cells were transiently transfected (24 h) using Lipofectamine™ 3000 (L3000015, ThermoFisher Scientific, USA) according to the manufacturer information. Plasmids used for the co-transfection are FLAG-IKK-β (NM_001556, RC219154, Origene, USA) and KCTD15 (NM_024076.2, EX-A3845-M67, TebuBio, Italy). A 2 mg of co-transfected HeLa cell extract was incubated with 2 μg/mL of anti-FLAG (F1804, Sigma Aldrich) antibody for 4 h at 4 °C. For each experiment (endogenous and recombinant immunoprecipitations) 75 µL of Protein A/G beads (sc-2003, Santa Cruz Biotechnology, Dallas, TX) in Tris–HCl 20 mM pH 7.4 + NaCl 150 mM 0.1% BSA was added and incubated for 16 h at 4 °C. Beads were washed 5 times with Tris–HCl 20 mM pH 7.4 + NaCl 150 mM and boiled for 5 min in 50 μL Laemmli buffer. Samples were loaded on SDS-PAGE and subjected to Western blot analyses. Images were acquired with the ChemiDoc Imaging System (Bio-Rad, USA) coupled with Image Lab software.

### Fluorescence microscopy

RS4;11 and SEM smears were fixed using a cytology fixative (Bio-FIX 05-X200, Bio-Optica, Italy). SKBR3 were treated with 15 μM of JSH-23 NF-κB inhibitor (ab144824, abcam) for 3 h at 37 °C. Subsequently, cells were fixed using freeze (− 80 °C) methanol for 10 min. The slides were subjected to blocking with a solution of 3% (w/v) BSA in PBS pH 7.4 at Room Temperature. Anti NF-κB (Rabbit, 622602 Biolegend, USA) antibody was diluted 1:100 in a solution of PBS + 1% (w/v) BSA and then all slides were incubated for 4 h at + 4 °C. After five wash steps in PBS for 5 min each, FITC-conjugated anti-rabbit secondary antibody (F0382, Sigma Aldrich) diluted 1:200 in a solution of PBS + 1% (w/v) BSA was incubated for 1 h at 4 °C in the dark. For SKBR3 an anti β-tubulin (1:100) followed by a PE- conjugated anti-mouse secondary antibody (1:200, A10543, Thermo Fischer) were added to detect cytoplasm. After an additional five wash steps in PBS, a solution of 4′,6-Diamidino-2-Phenylindole, Dihydrochloride (DAPI, Thermo Fischer Scientific D1306) diluted 1:35,000 in PBS was used for coloring of the nuclei.

Images were obtained using an automated upright microscope system (Leica DM5500 B) coupled with Leica Cytovision software.

### Flow cytometry experiments

Flow cytometry experiments with a minimum of 10.000 recorded events were performed using the Cytomics FC500 and the Cytoflex V2-B4-R2 (Beckman-Coulter, CA, USA). Routine control of instrument sensitivity was performed and no change in instrument sensitivity was seen throughout the study. Intracellular or combined intracellular plus surface staining was performed by the use of the PerFix Expose kit (B26976, Beckman Coulter), following the manufacturer’s instruction. Data were analyzed using Kaluza analysis software version 2.1 (Beckman-Coulter, CA, USA). The following monoclonal antibodies were used for the FCM experiments: anti KCTD15 (GTX50002, Genetex International, USA), anti pIKK-α/βS176/177 J.10.3, Thermo Scientific, USA), pIKB-α S32/36 (H.709.9 Thermo Scientific, USA) and anti IKB-α (662402, Biolegend, USA). FITC- conjugated anti-mouse (ab7064, Abcam, UK) or anti-rabbit (F0382, Sigma Aldrich) secondary antibodies were used for protein detection.

Proximity Ligation Assay (PLA) employs a pair of oligonucleotide-conjugated antibodies (called PLA probes) with an affinity for the primary antibodies that will be used. If two targeted proteins can be recognized by the primary antibodies from two different species and can interact, the PLA probes will remain nearby. In this way, the PLA probes serve as templates for the hybridization of two additional DNA oligonucleotides, guiding their ligation into DNA circles. The circles will be locally amplified by rolling circle amplification to generate intracellular fluorescent products that will be studied by FCM^52^. For these experiments, 5 × 10^5^ RS4;11 and SEM cells were fixed and permeabilized using PerFix Expose Kit. Protein interaction were detected using the following antibody pairs: (i) Anti IKB-α (662402, Biolegend, USA, Mouse) with Anti NF-κB (622602 Biolegend, USA, Rabbit,); (ii) anti-IKK-β (GTX107970, Genetex International, USA, Rabbit) with Anti KCTD15 (GTX50002, Genetex International, USA, Mouse). Primary monoclonal antibodies were added at a concentration of 1:50 in Buffer 3. After 30 min of incubation 2 wash steps in PBS 1 × were performed and PLA protocol was applied according to the manufacturer’s instructions (DUO94002, DUO92001, DUO92005, Sigma Aldrich, Germany). PLA assays were acquired on Cytoflex cytofluorimeter and fluorescence was recorded in the FITC channel.

### Statistical analysis and reproducibility

p-values were calculated as described in individual figure legends using GraphPad Prism 7 (GraphPad Software). Numbers of biological and/or technical replicates as well as a description of the statistical parameters are stated in the figure legends. All experimental images are representative of at least two independent experiments.

### RNA-sequencing and in silico analyses

Gene expression levels from previous RNA-Seq results^9^, were retrospectively used for functional genomic analyses using the Ingenuity Pathway Analysis software (IPA, QIAGEN Inc., https://www.qiagenbioinformatics.com/products/ingenuitypathway-analysis). The top statistically significant IPA canonical pathways with enrichment score threshold (− log adj p-value) ≥ 5, by using Benjamini–Hochberg approach for Multiple Testing Correction^50^ were ranked for the relative ratio of differentially expressed genes overlapping the total number of molecules within each Ingenuity Knowledgebase pathways (n. 7). We carried out in silico prediction analyses of the KCTD15 promoter region and RELA/NF-κB TF binding sites by NCBI database^51^ and Contra V3 web server^52^. We analyzed the KCTD15 promoter region (1000 bp and 500 bp upstream) with respect to the reference sequences (RefSeq) “NM_001129994.2” and “NM_001129995.2”. We carried out a positional weight matrices (PWMs) approach^53^ with stringency parameters core = 0.95, similarity matrix = 0.85.

## Supplementary Information

Supplementary Information.

## Data Availability

The datasets analyzed during the current study are available in the Bio project: PRJNA601326 (more information at reference number 9).

## Abbreviations

NF-κB: Nuclear factor kappa-light-chain-enhancer of activated B cells
IKB-α: Inhibitor α of NF-κB
IKK-β: IKB-α kinase
HSPC: Hematopoietic stem and progenitor cell
MLL: Mixed-lineage leukemia
ALL: Acute lymphoid leukemia
AML: Acute myeloid leukemia
PLA: Proximity ligation assay
PBMC: Peripheral blood mononuclear cells
2′F-ANA-scrambled: 2′-Deoxy, 2′Fluroarabino Nucleic Acids scramble sequence
2′F-ANA-KCTD15: 2′-Deoxy, 2′Fluroarabino Nucleic Acids against KCTD15 mRNA
IPA: Ingenuity pathway analysis
FCM: FlowCytoMetry.
KCTD: (K)potassium channel tetramerization domain containing protein
