# Supplementary material for: KCTD15 deregulation is associated with alterations of the NF-κB signaling in both pathological and physiological model systems
Source: Sci Rep. 2021 Sep 14;11:18237. doi: 10.1038/s41598-021-97775-6 (PMC8440651; doi:10.1038/s41598-021-97775-6)
Supplement: Supplementary file 1 — Supplementary Information. [file 41598_2021_97775_MOESM1_ESM.pdf]

TITLE

KCTD15 deregulation is associated with alterations of the NF-κB signaling in both pathological and physiological model systems

AUTHORS

Giovanni Smaldone[1], Luigi Coppola[1], Katia Pane[1], Monica Franzese[1], Giuliana Beneduce[2], Rosanna Parasole[2], Giuseppe Menna[2], Luigi Vitagliano*[3], Marco Salvatore[1] and Peppino Mirabelli*[1]

Correspondence to: * Dr. Luigi Vitagliano (luigi.vitagliano@unina.it)

*Dr. Peppino Mirabelli (peppino.mirabelli@synlab.it)

AFFILIATIONS

[1]IRCCS SDN, Napoli, Via E. Gianturco 113, 80143 Naples, Italy.

[2] Department of Pediatric Hematology-Oncology, Santobono-Pausilipon Hospital, 80129 Naples, Italy

[3] Institute of Biostructures and Bioimaging, C.N.R., Via Mezzocannone n.16, 80134 Napoli, Italy.

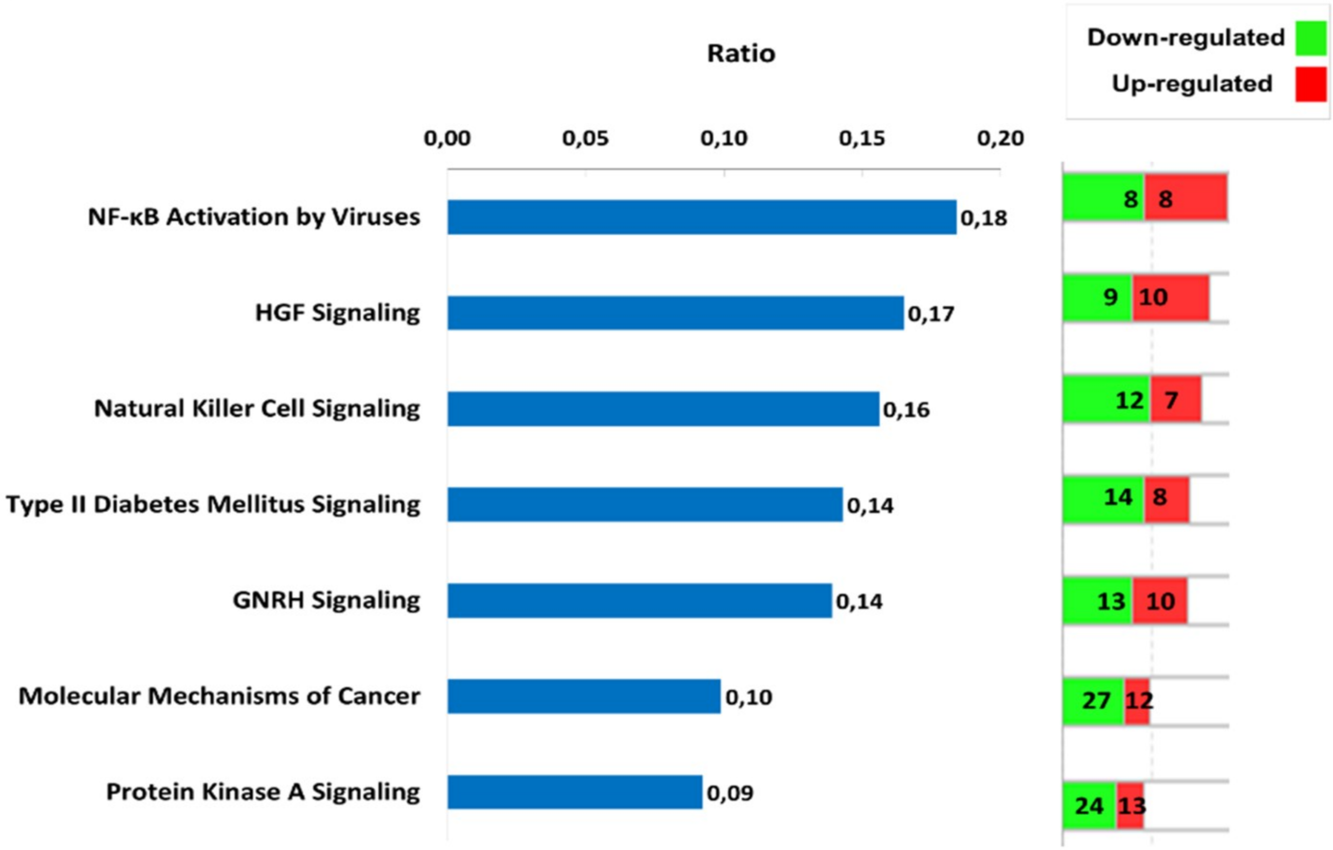

Supplementary figure 1. NF-κB signalling is the most enriched pathway according to Ingenuity Pathway Analysis (IPA). Top pathways (left side) with enrichment score greater than 5 [-log (B-H p value)] are ranked by the ratio of overlapping DE genes with respect to the total number of molecules that define each pathway. The relative number of down- regulated (green) and up-regulated (red) genes within each canonical pathway (bolded numbers) are showed in the bar-chart on the right

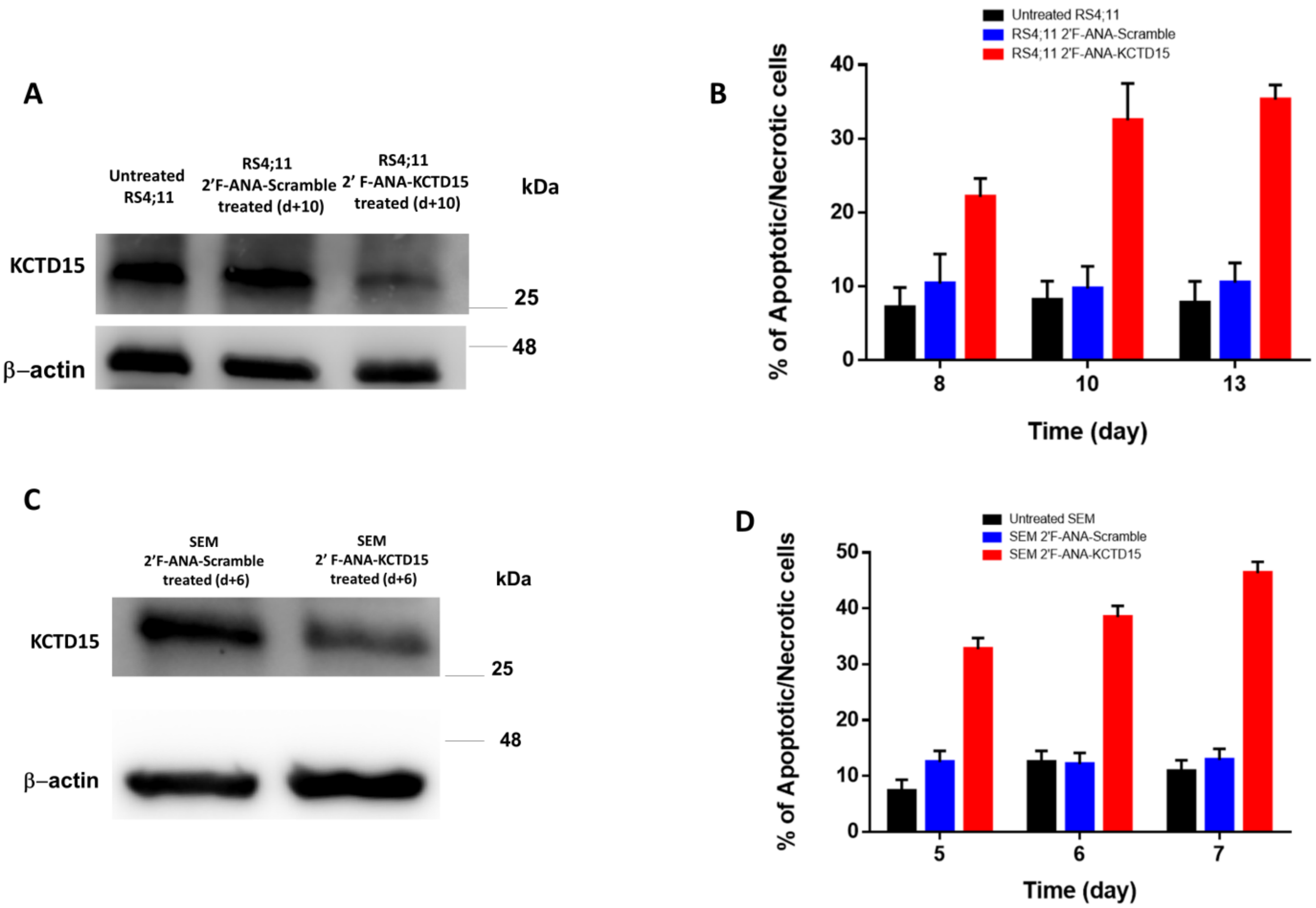

Supplementary Figure 2. KCTD15 silencing causes death in B-ALL cell lines. Western blot showing endogenous expression of KCTD15 protein in RS4;11 (A) and SEM (C) cells untreated as well as treated with 2'F-ANA Scramble and 2'F-ANA KCTD15 at day +10 and + 6 respectively. β-actin was used as internal control; number represent molecular weight of protein marker expressed in kDa. Histogram plots report the % of necrotic/apoptotic cells during the treatment with 2'F-ANA-KCTD15 in comparison with untreated and 2'F-ANA Scamble treated RS4;11 (B) and SEM (D). % of necrotic/apoptotic cells is shown as mean +/- SD of three technical independent experiments

RS4;11

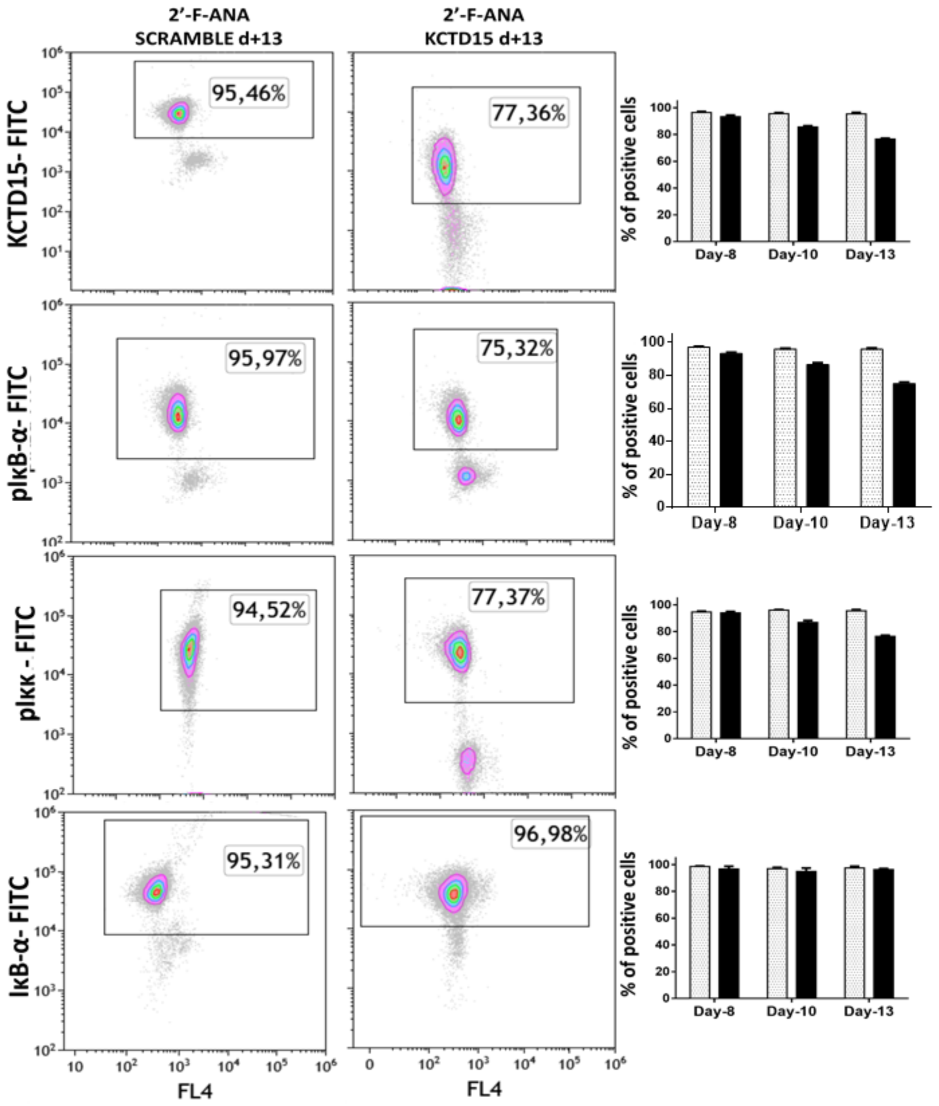

Supplementary Figure 3. Density-plots show KCTD15, pIκβ-α, pIκκ-β, and IκB-α expression, in terms of percentage of positive cells, at day+13 in RS4;11. Bar-histograms show the complete time-course analysis of treatment for 2'F-ANA Scramble (checkerboard bars) and 2'F-ANA KCTD15 (black bars). % of positive cells is shown as mean +/- SD of three technical independent experiments

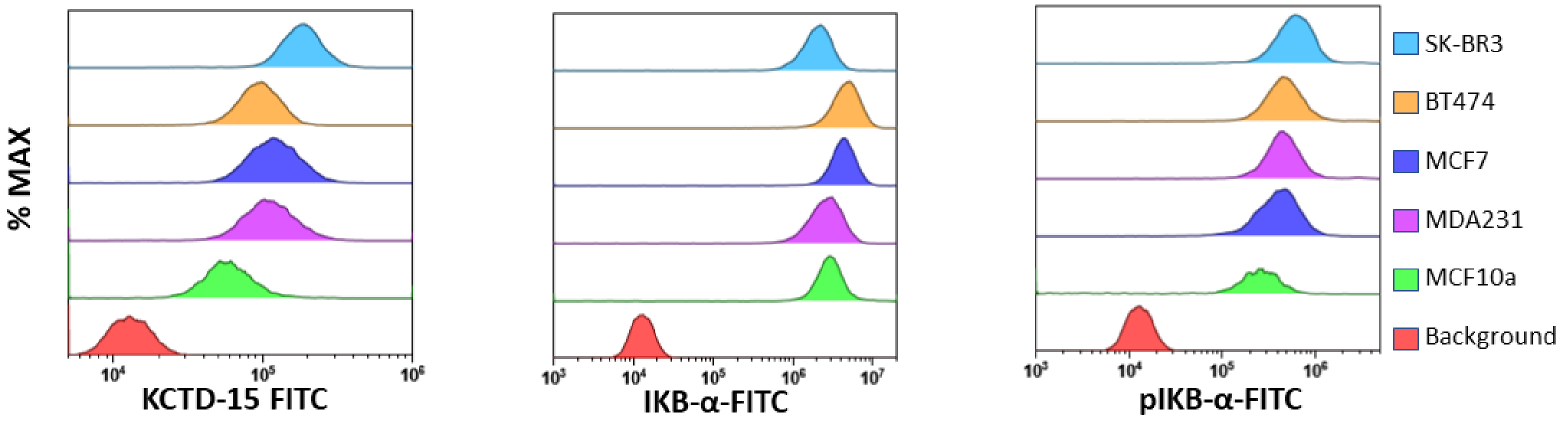

Supplementary Figure 4. Overlay histograms of KCTD15 (left), IKB-α (middle) and pIKB-α (right) protein levels in 5 different breast cancer cell lines. SKBR3 (light blu) cell line was selected as a model system featured by high KCTD15 expression and active NF-kB pathway due to the increased pIKB-α levels and low IKB-α total expression in comparison to other breast cancer cell lines..

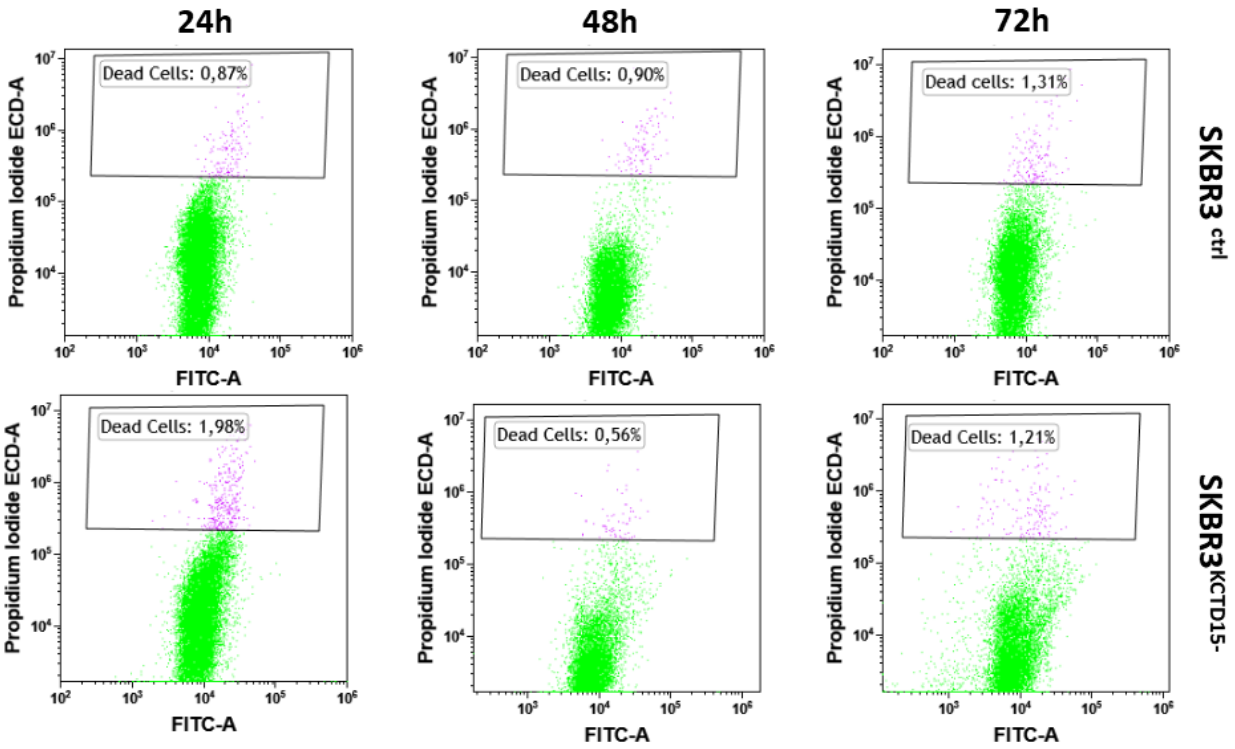

Supplementary Figure 5. SKBR3^{ctrl} and SKBR3$^{KCTD15-}$ cell line vitality at three different time; 24h (left), 48h (middle) and 72h (right). KCTD15 gene was inactivated by CRISP/CAS9 in SKBR-3 cell line and a stable clone was generated in culture after selection with puromycin. The Cellular viability was not impaired after KCTD15 loss. Numbers represent the percentage of Propidium iodide positive cells.

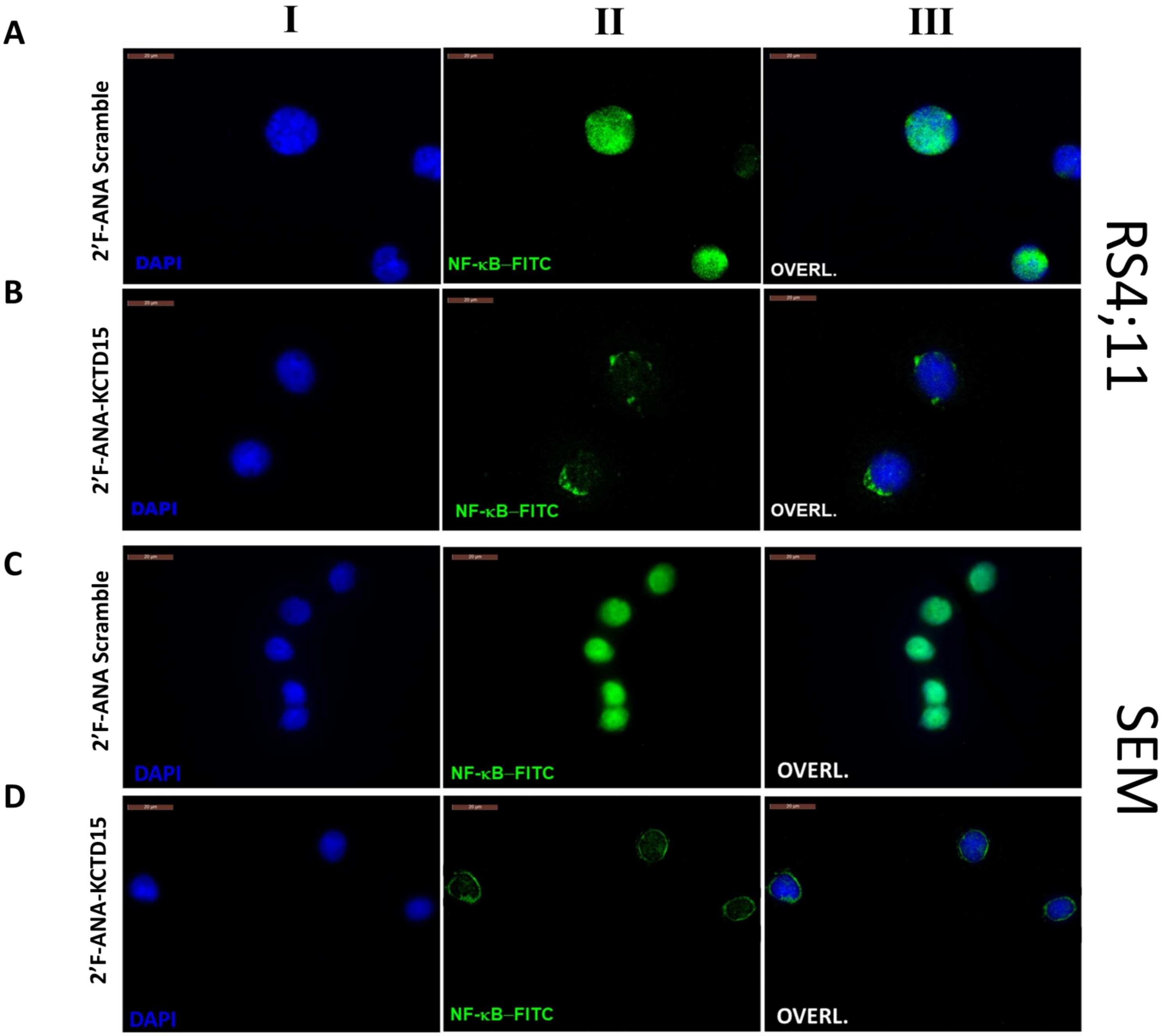

Supplementary Figure 6. Immunofluorescence experiments at day+13 (RS4;11, panel A and B) and day +7 (SEM, panel C and D) showing NF-κB cytoplasmic localization after treatment with 2'F-ANA KCTD15 in comparison to 2'F-ANA Scramble treated cells. Column I) Nuclei staining with DAPI (blue). Column II) NF-κB (RelA)-FITC fluorescence (green). III) Overlapping of FITC and DAPI channels. Magnification 63x. Scale bars 20μm.

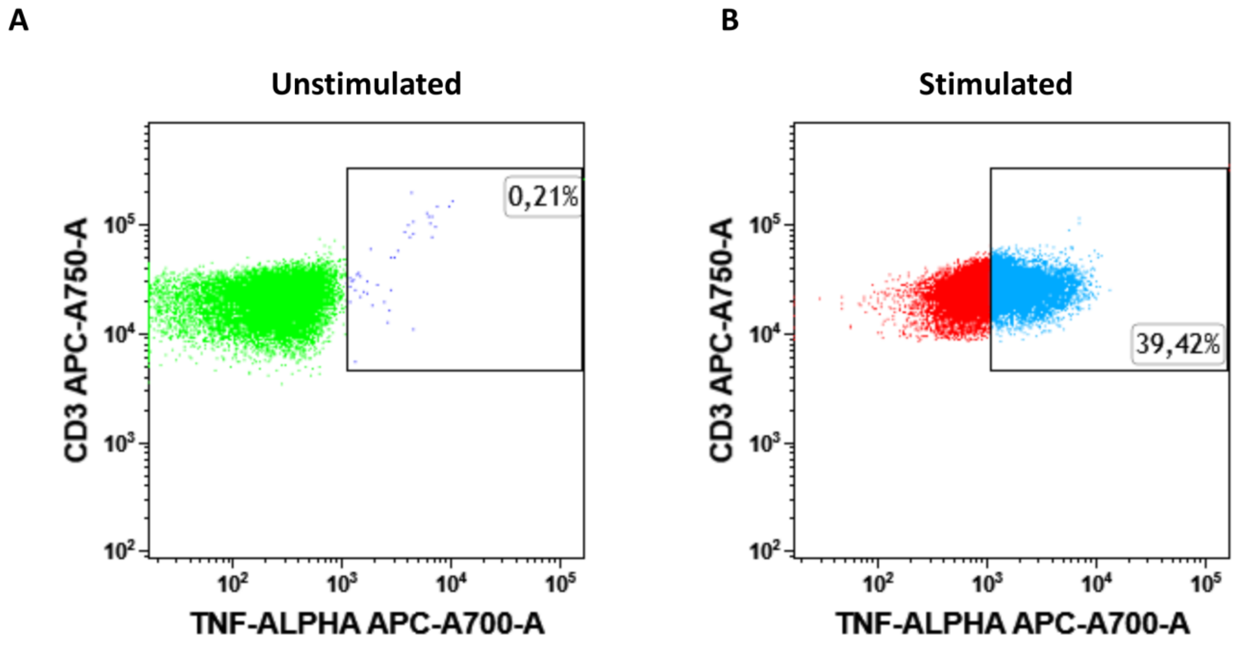

Supplementary Figure 7. Stimulated lymphocytes over-express TNF-α. CD3-APC750 vs TNFα-APC700 dot plots of unstimulated (A) and DurActive Stimulated (B) lymphocytes. Numbers report percentage of gated cells. Experiments were repeated twice with similar results.

.

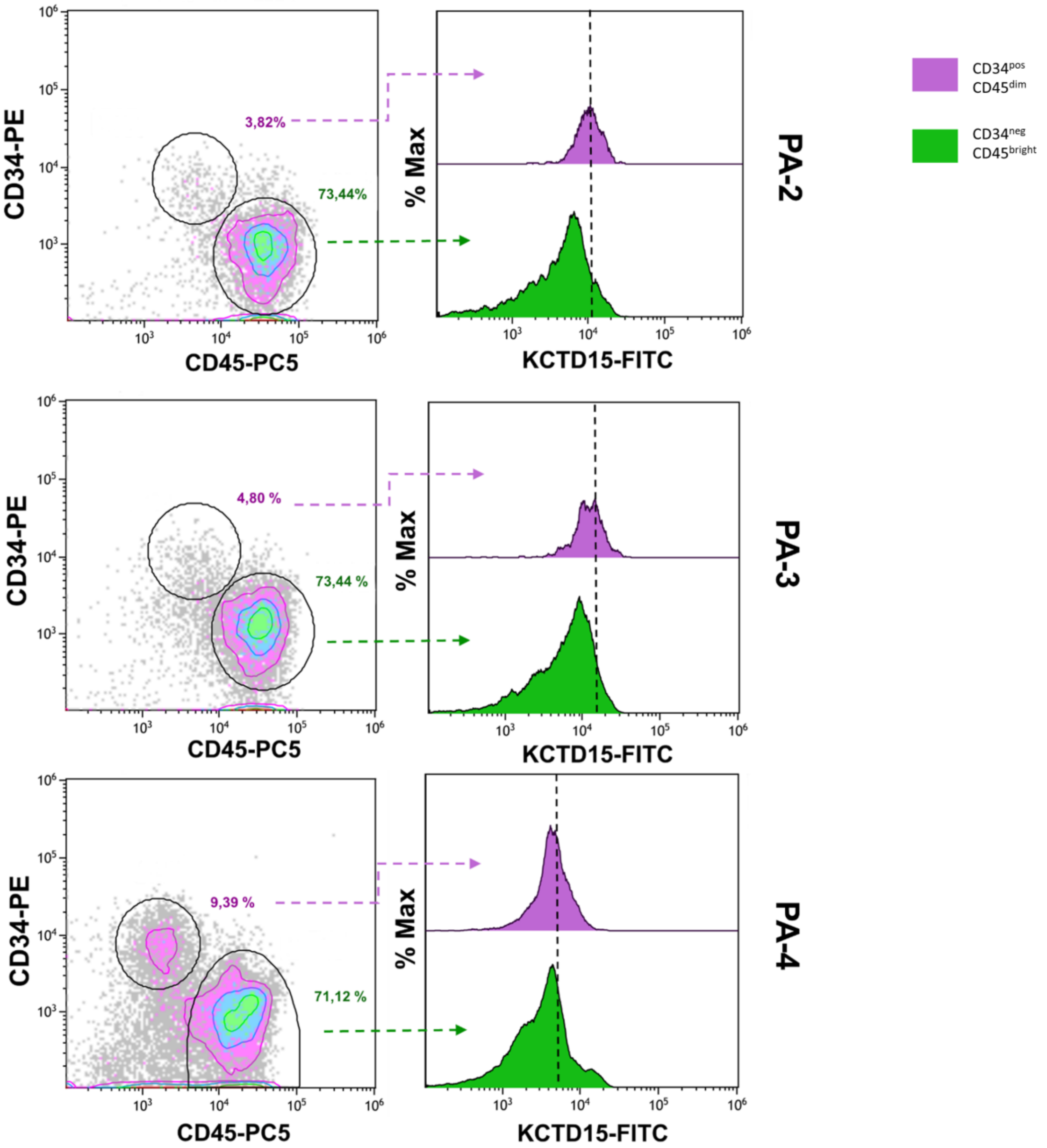

Supplementary Figure 8. KCTD15 is over-expressed in hematopoietic stem cells. We performed a combined intracellular and surface FCM analysis in 3 exemplificative cases of pediatric B-ALL patients after therapy at day +33. Contour with density plot showing selection of hematopoietic stem cells ($CD34^{pos}/CD45^{dim}$, violet events) and mature cells ($CD34^{neg}/CD45^{pos}$, green events) in density gradient purified BM cells. Numbers are referred to the percentage of gated cells. Overlay histograms showing the higher levels of KCTD15 in the $CD34^{pos}/CD45^{dim}$ (violet) stem cells compared to $CD34^{neg}/CD45^{pos}$ (green) cells. PA-# patient # after treatment at day +33.

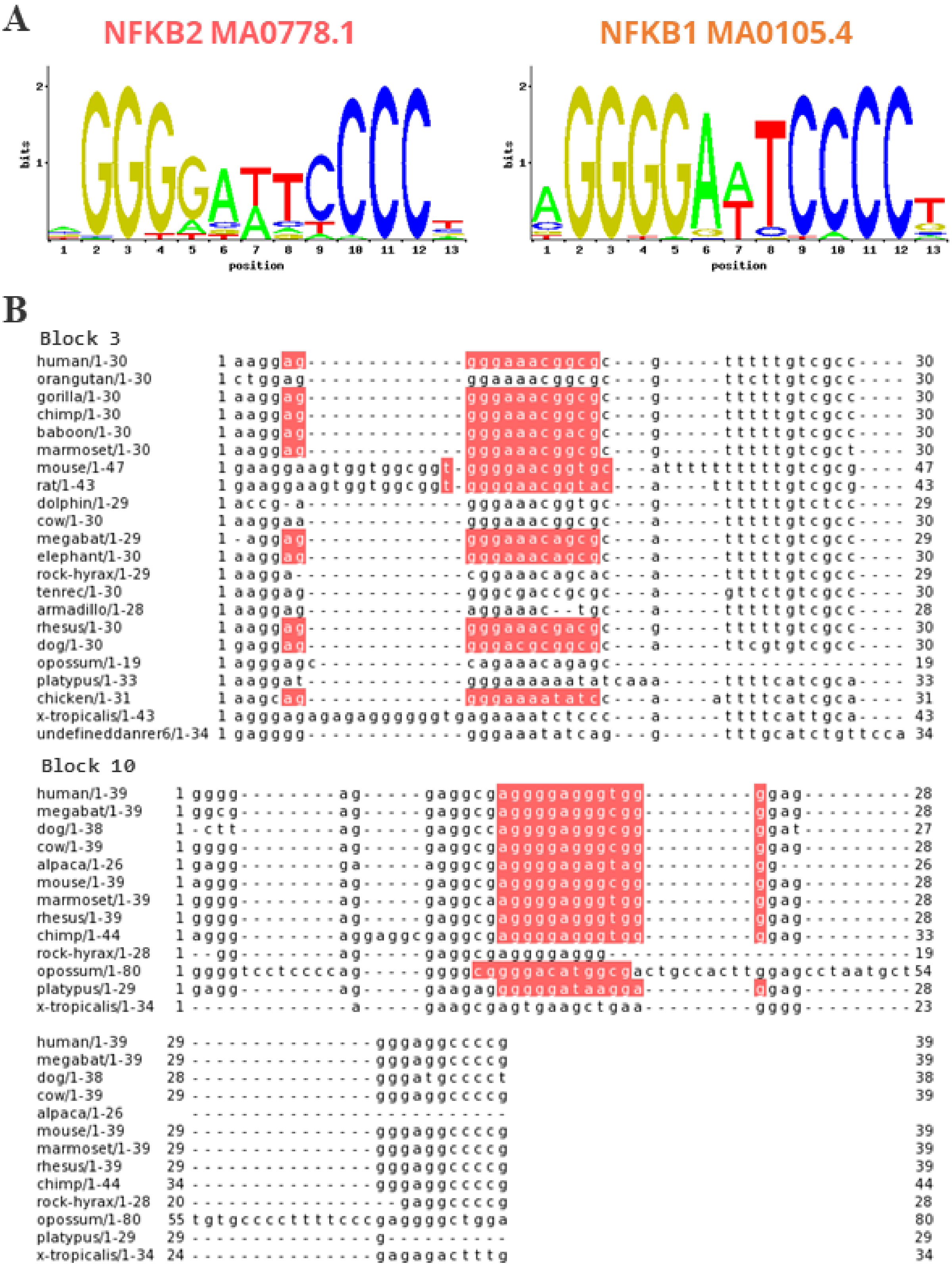

Supplementary Figure 9. In silico prediction analysis of NFKB and RELA family transcription factor binding sites in 500 bp upstream KCTD15 promoter gene TSS chr19:34287750. A - Logos of the two top-scoring transcription factor consensus binding sites (NFKB1, ID MA0105.4 and NFKB2, MA0778.1) by Contra V3. B - Visualization of alignment of promoter block nr. 3 position: chr19:34287301-34287331 and block nr. 10 position chr19:34287643-34287682 respectively.

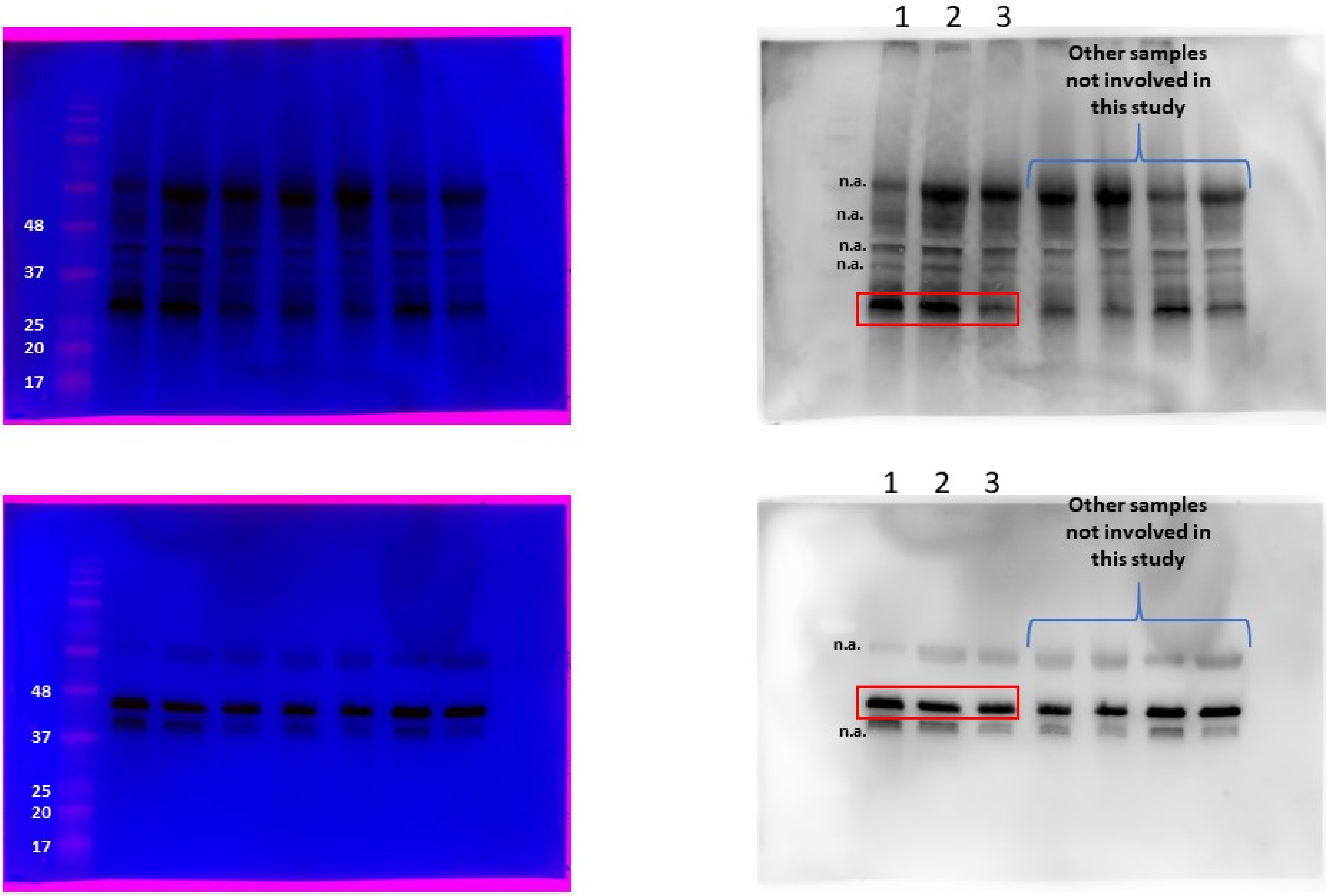

Supplementary Figure 10. Entire WB of Supplementary Figure 2A. Western blot showing whole cell extract of Untreated RS411 (lane 1), 2'F-ANA Scramble Treated RS4;11 at day10 (2) and 2'F-ANA-KCTD15 Treated RS4;11 at day10 (3).. Numbers represent molecular weight of protein marker expressed in kDa. To the left multichannel acquisition to see protein marker. To the right chemiluminescent acquisition to see target proteins; Upper panel reports acquisition using anti-KCTD15 antibody; lower panel reports acquisition using anti-Actin antibody. Red square= portion of western blot showed in the Supplementary Figure 2A. n.a.= not assigned bands

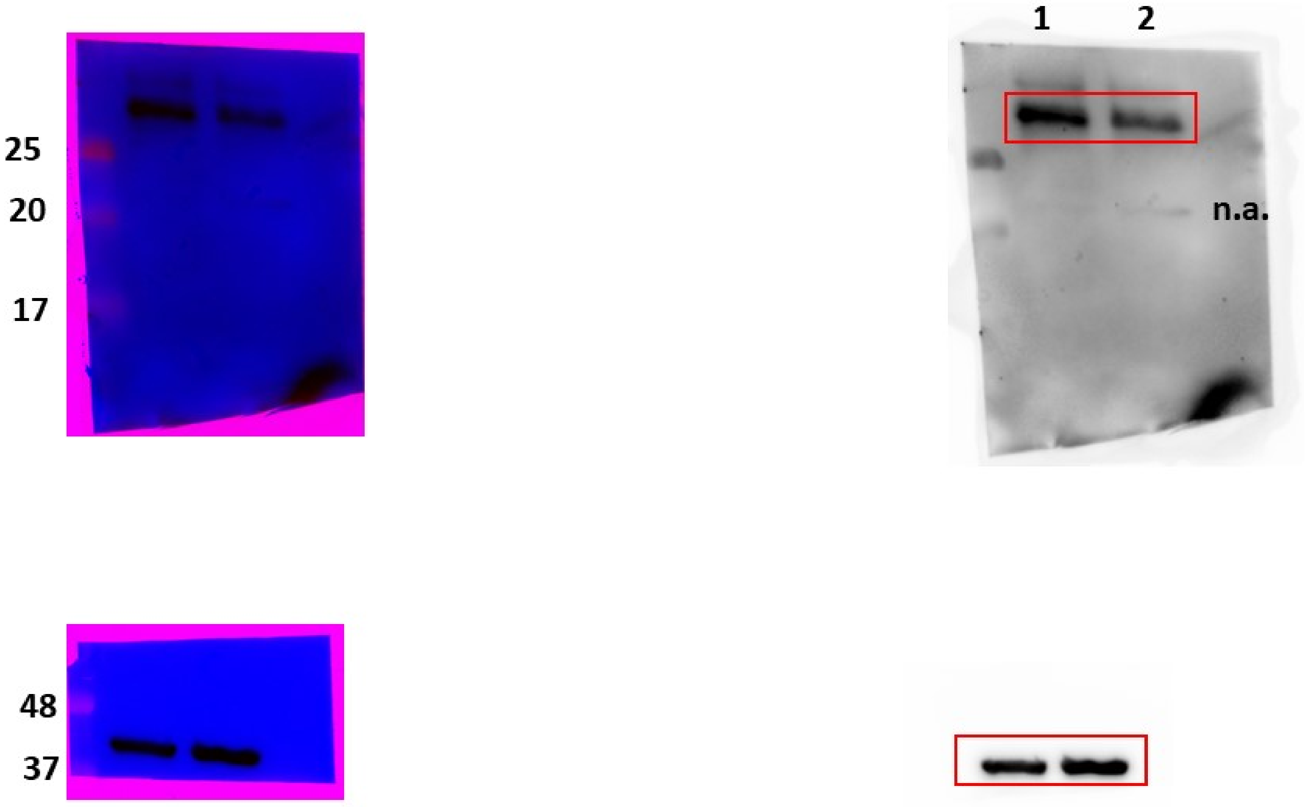

Supplementary Figure 11. Entire WB of Supplementary Figure 2C. Western blot showing whole cell extract of 2’F-ANA Scramble Treated SEM at day6 (1) and 2’F-ANA-KCTD15 Treated SEM at day6 (2). Numbers represent molecular weight of protein marker expressed in kDa. To the left multichannel acquisition to see protein marker. To the right chemiluminescent acquisition to see target proteins; Upper panel reports acquisition using anti-KCTD15 antibody; lower panel reports acquisition using anti-Actin antibody. Red square= portion of western blot showed in the Supplementary Figure 2C. n.a.= not assigned bands

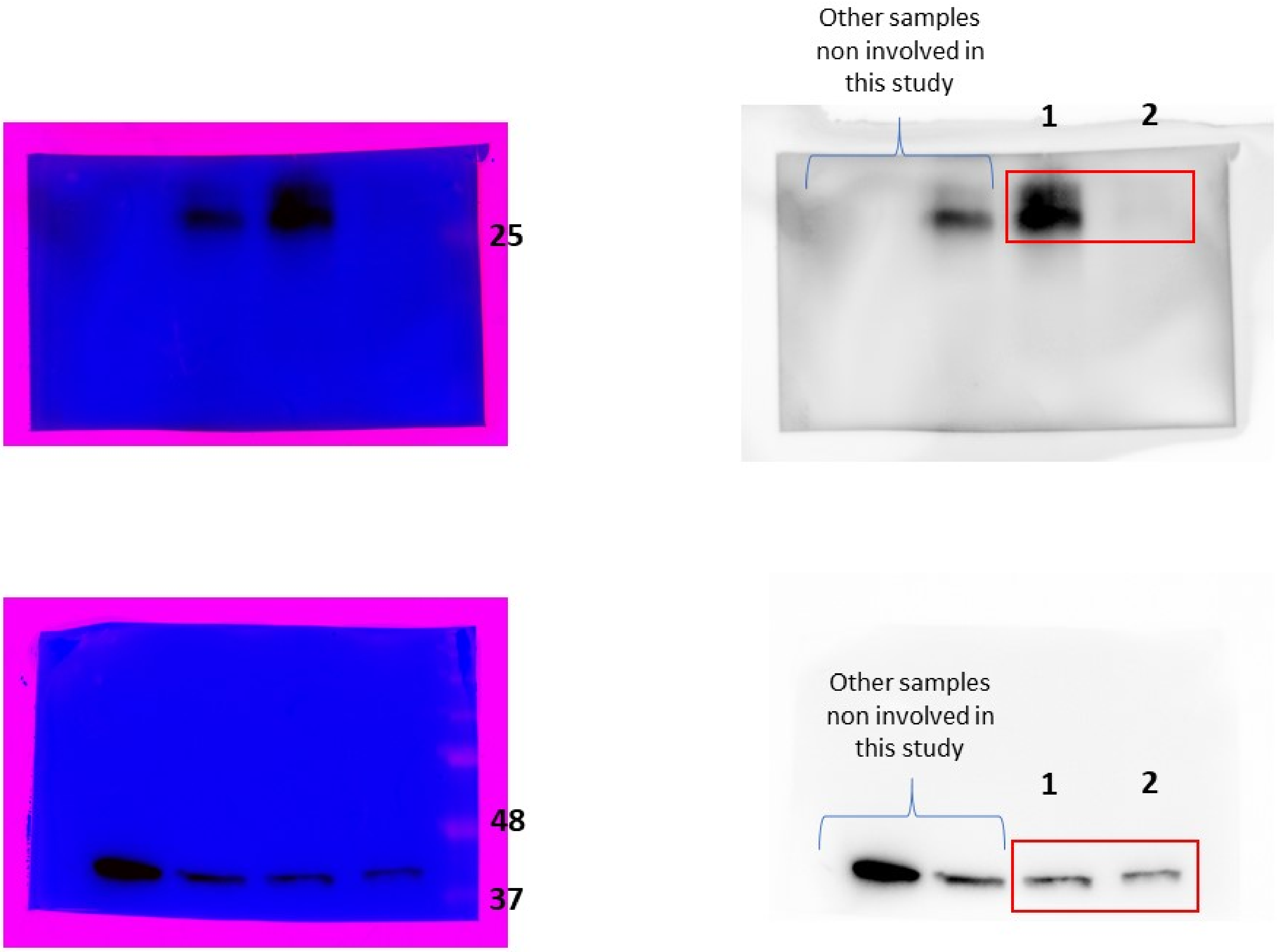

Supplementary Figure 12. Entire WB of Figure 2A. Western blot showing whole cell extract of SKBR3^{CTRL} (1) and SKBR3$^{KCTD15-}$ (2). Numbers represent molecular weight of protein marker expressed in kDa. To the left multichannel acquisition to see protein marker. To the right chemiluminescent acquisition to see target proteins; Upper panel reports acquisition using anti-KCTD15 antibody; lower panel reports acquisition using anti-Actin antibody. Red square= portion of western blot showed in the Figure 2A.

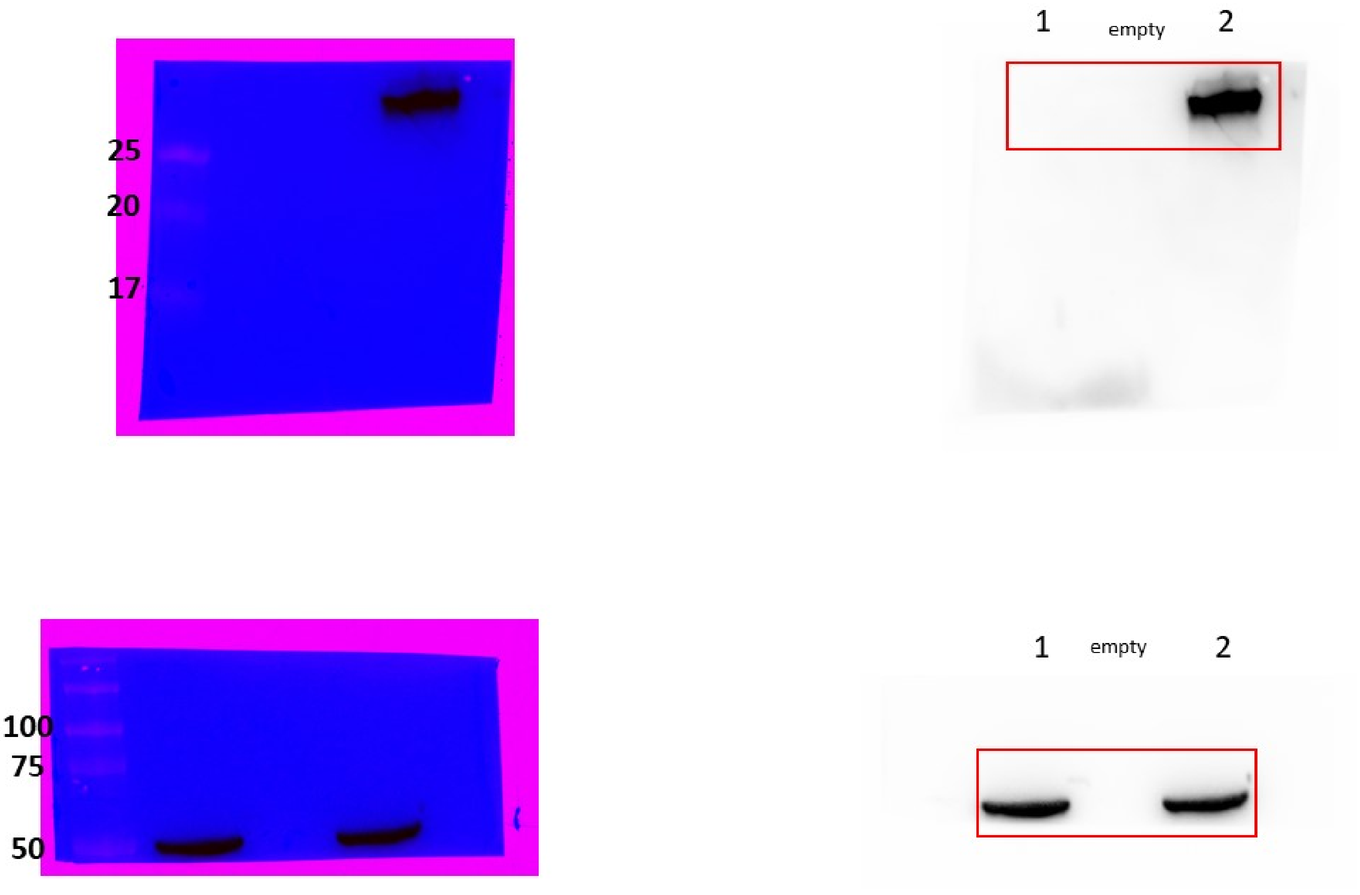

Supplementary Figure 13. Entire WB of Figure 3B. Western blot showing whole cell extract of NF-κB reporter (Luc) - HEK293 cells (1) and NF-κB reporter (Luc) - HEK293 over-expressing FLAG-KCTD15 (2). Numbers represent molecular weight of protein marker expressed in kDa. To the left multichannel acquisition to see protein marker. To the right chemiluminescent acquisition to see target proteins; Upper panel reports acquisition using anti-FLAG antibody; lower panel reports acquisition using anti-Tubulin antibody. Red square= portion of western blot showed in the Figure 2A.

IP IKB-α

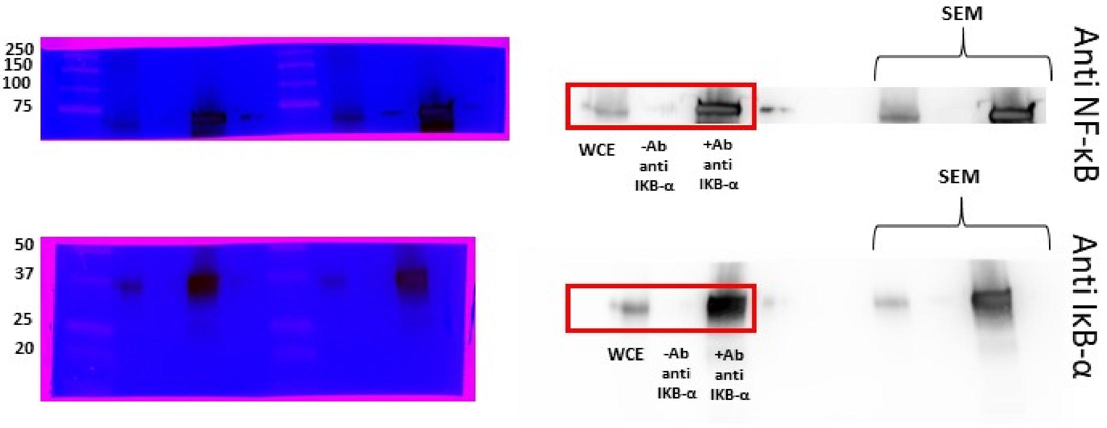

IP Iκκ-β su RS4;11

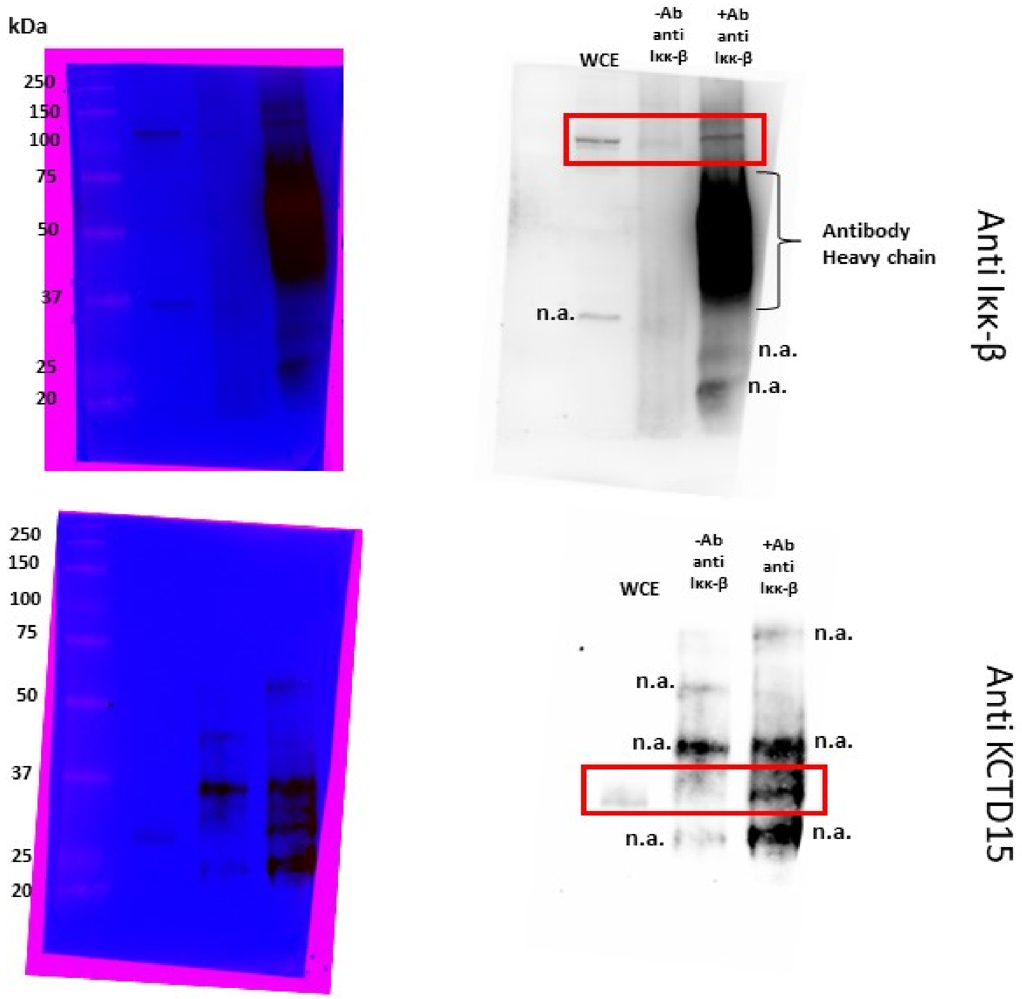

Supplementary Figure 14. Entire WB of Figure 7A. Western blot showing immunoprecipitation of IKB-α (upper panels) and IKK-β (lower panels) in RS4;11 cells. Numbers represent molecular weight of protein marker expressed in kDa. To the left multichannel acquisition to see protein marker. To the right chemiluminescent acquisition to see target proteins. Red square= portion of western blot showed in the Figure 7A of Main text. WCE= whole cell extract. n.a.= not assigned bands.

IP IKB-α

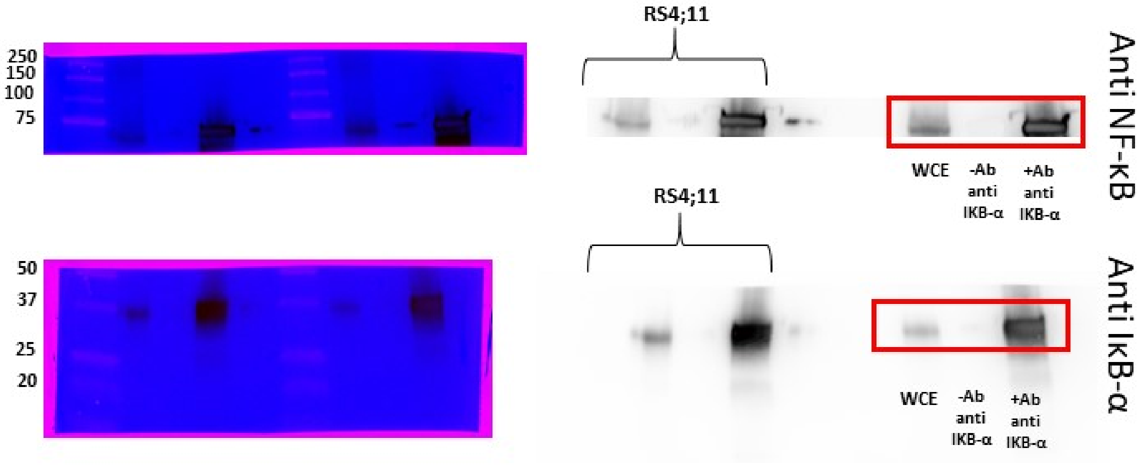

IP Iκκ-β su SEM

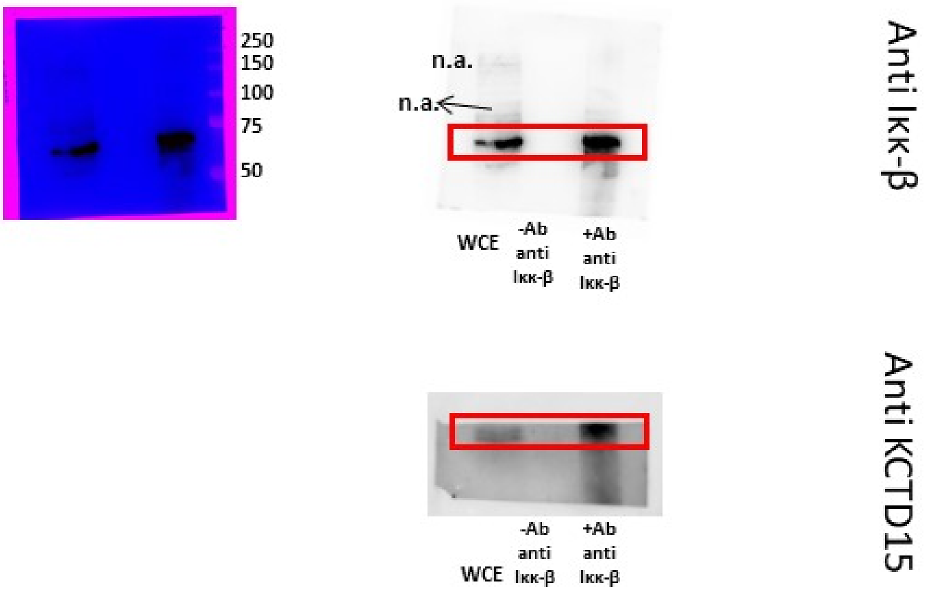

Supplementary Figure 15. Entire WB of Figure 7B. Western blot showing immunoprecipitation of IκB-α (upper panels) and Iκκ-β (lower panels) in SEM cells. Numbers represent molecular weight of protein marker expressed in kDa. To the left channel multichannel acquisition to see protein marker. To the right chemiluminescent acquisition to see target proteins. Red square = portion of western blot showed in the Figure 7B of Main text. WCE= whole cell extract. n.a.= not assigned bands.

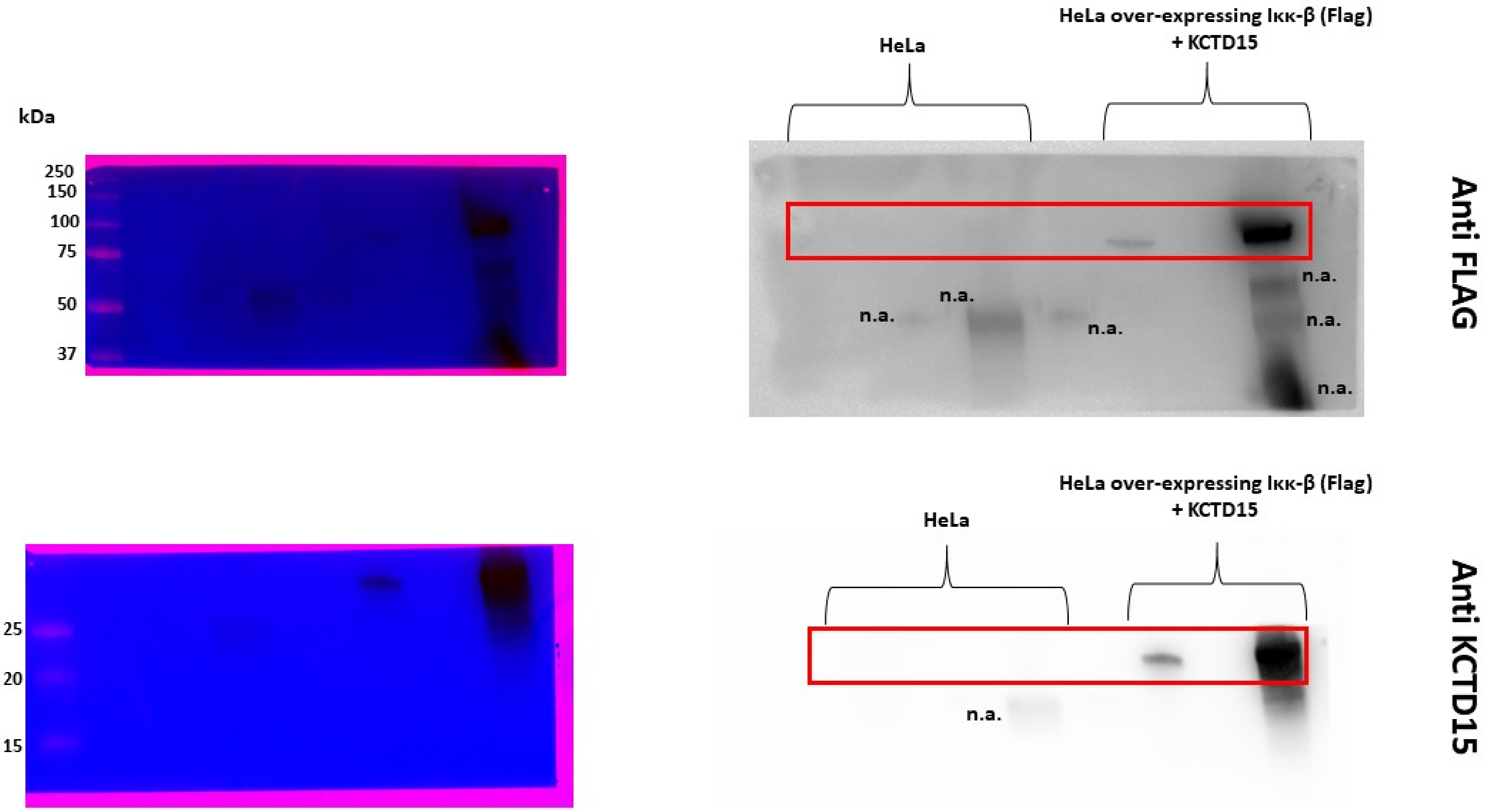

Supplementary Figure 16. Entire WB of Figure 7D. Western blot showing immunoprecipitation of FLAG-IKK-β in HeLa over expressing FLAG-IKK-β and KCTD15. Numbers represent molecular weight of protein marker expressed in kDa. To the left channel multichannel acquisition to see protein marker. To the right chemiluminescent acquisition to see target proteins. Upper panel reports acquisition using anti-FLAG antibody; lower panel reports acquisition using anti-KCTD15 antibody. Red square = portion of western blot showed in the Figure 7D of Main text. n.a.= not assigned bands.
